# Oxyresveratrol: Sources, Productions, Biological Activities, Pharmacokinetics, and Delivery Systems

**DOI:** 10.3390/molecules26144212

**Published:** 2021-07-11

**Authors:** Kittisak Likhitwitayawuid

**Affiliations:** Department of Pharmacognosy and Pharmaceutical Botany, Faculty of Pharmaceutical Sciences, Chulalongkorn University, Bangkok 10330, Thailand; Kittisak.L@chula.ac.th

**Keywords:** oxyresveratrol, synthesis, culture, tyrosinase, antioxidant, antiviral, neuroprotective, anticancer, metabolism, delivery system

## Abstract

Oxyresveratrol has recently attracted much research attention due to its simple chemical structure and diverse therapeutic potentials. Previous reviews describe the chemistry and biological activities of this phytoalexin, but additional coverage and greater accessibility are still needed. The current review provides a more comprehensive summary, covering research from 1955 to the present year. Oxyresveratrol occurs in both gymnosperms and angiosperms. However, it has never been reported in plants in the subclass Sympetalae, and this point might be of both chemotaxonomic and biosynthetic importance. Oxyresveratrol can be easily obtained from plant materials by conventional methods, and several systems for both qualitative and quantitative analysis of oxyresveratrol contents in plant materials and plant products are available. Oxyresveratrol possesses diverse biological and pharmacological activities such as the inhibition of tyrosinase and melanogenesis, antioxidant and anti-inflammatory activities, and protective effects against neurological disorders and digestive ailments. However, the unfavorable pharmacokinetic properties of oxyresveratrol, including low water solubility and poor oral availability and stability, have posed challenges to its development as a useful therapeutic agent. Recently, several delivery systems have emerged, with promising outcomes that may improve chances for the clinical study of oxyresveratrol.

## 1. Introduction

Oxyresveratrol (2,3′,4,5′-tetrahydroxystilbene) is one of the natural stilbenes that has recently received much attention due to its simple chemical structure and diverse therapeutic potentials. Previous reviews describe the chemistry and biological activities of this phytoalexin [1,2], but additional coverage and greater accessibility are necessary.

**Figure 1:**
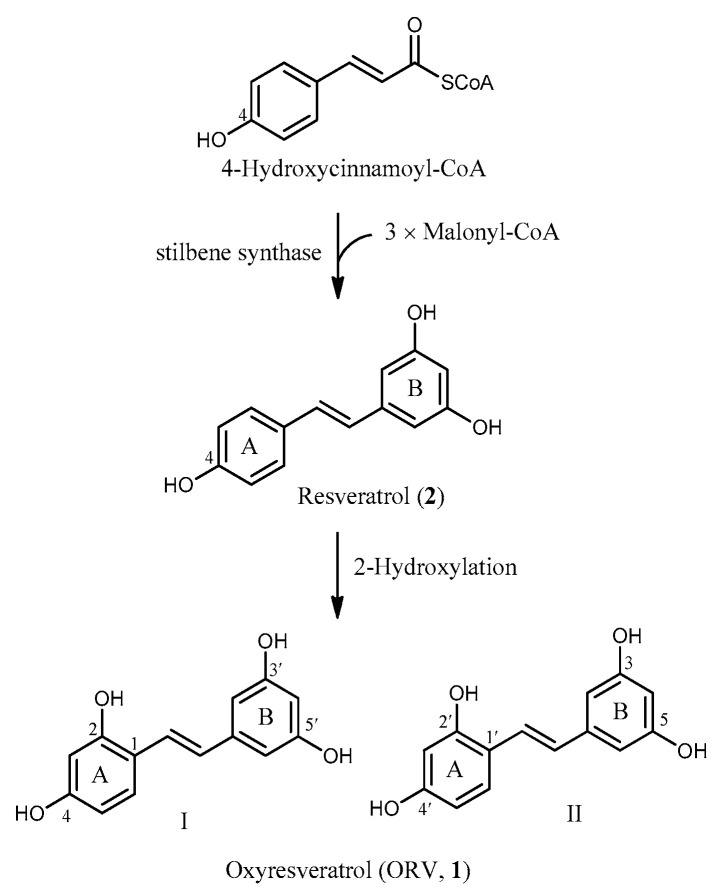
Proposed biosynthesis of oxyresveratrol (ORV, **1**).

The structure of oxyresveratrol (systematic name: 4-[(*E*)-2-(3,5-dihydroxyphenyl)-ethenyl] benzene-1,3-diol; C_14_H_12_O_4_) contains a *trans*-1,2-diphenylethylene nucleus with two OH groups on each of the aromatic rings (Figure 1).

Oxyresveratrol (ORV, **1**) is believed to be derived from resveratrol (**2**), a better-known polyhydroxystilbene [3,4,5], but no experimental evidence has yet been obtained. The A-ring and ethylene bridge carbons are assumed to come together from 4-hydroxycinnamoyl-CoA, and the B ring carbons are from three units of malonyl-CoA through the mixed shikimate-acetate pathway. The Aldol reaction of the starting molecules occurs to yield resveratrol after decarboxylation. Hydroxylation at C-2 of resveratrol generates ORV [5]. In the literature, two different carbon numbering systems for ORV (I and II) are in use (Figure 1), and this, at times, leads to confusion, particularly when the positions of the OHs are indicated. This review adopts the first system (I), in line with most reports.

**Figure 2:**
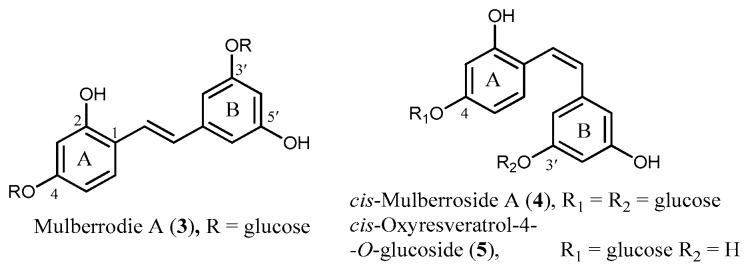
Structures of some glucosidic ORVs.

ORV (**1**) occurs in both free and glycosidic forms. Although the compound has four OH groups, it has not been reported in the form of a tri- or tetra-*O*-glycoside. Until recently, only mono- and di-*O*-glucosidic ORVs have been isolated and characterized. Mono-*O*-glucosylated ORVs have been found to exist as a 2-, 4- or 3′-*O*-glucoside [6,7,8,9,10,11,12,13,14]. The only natural di-glucosidic ORV identified so far is oxyresveratrol 3′,4-diglucoside, most often known as mulberroside A (**3**) [15] (Figure 2). Some glycosidic ORVs have a *cis* configuration; for example, *cis*-mulberroside A (**4**) and *cis*-Oxyresveratrol 4-glucoside (**5**) [15,16,17] (Figure 2). However, natural ORV in a free form is found only in the *trans* configuration.

## 2. Natural Sources

This review focuses on the natural occurrence of free ORV, as summarized in Table 1. These data may at first seem to suggest that ORV has a wide distribution in the plant kingdom, ranging from the gymnosperms to the angiosperms, but a thorough analysis of the records reveals that the compound indeed occurs sporadically and only in certain plant families.

In gymnosperms, ORV was reported from just one single plant, *Gnetum hainensis* (Gnetaceae) [21] (Table 1), although its oligomers were found in several species of *Gnetum* [18,19,20,21,22,23,24,25].

As for angiosperms, ORV occurs in only four families of the class Monocotyledons, i.e., Poaceae, Liliaceae, Smilacaceae, and Melanthiaceae. It should be noted that the last three families are in the same order, Liliales [127].

In the class Dicotyledons, ORV is present only in the subclasses Apetalae (Monochlamydeae) and Polypetalae, particularly in the families Moraceae, Myrtaceae, Rutaceae, Rosaceae, Vitaceae, Fabaceae, and Caesalpiniaceae. In the Moraceae, ORV is concentrated in two genera, i.e., *Artocarpus* and *Morus*. Interestingly, ORV (**1**) is completely absent in the subclass Sympetalae (Gamopetalae), although resveratrol (**2**), its parent analog, is found in a few members of this taxon [127]. It can be seen that the uneven occurrence of ORV in these taxonomic groups is indeed parallel to the artificial classification of flowering plants. Thus, it could be hypothesized that all the sympetalous plants lack the enzyme that catalyzes the 2-hydroxylation reaction of resveratrol (Figure 1), and this may be taken as evidence for a monophyletic relationship among the members of the clade Asterids in the modern Angiosperm Phylogeny Group (APG) system.

### 2.1. Extraction, Isolation, and Identification

In most studies, ORV is extracted and isolated from plant materials by classical methods, consisting of two major steps, i.e., (*a*) solvent extraction and (*b*) chromatographic separation, with variations in detail. A few unconventional techniques are noted, such as microwave-assisted extraction (MAE) and ultrasonic-assisted extraction (UAE). For example, MAE was used to extract ORV from the twig and root of *Morus alba* [91,101,102,103], whereas the UAE technique was applied for the extraction of ORV from the bark and root of *Morus atropurpurea*, *Morus alba*, and *Morus latifolia* [92], and from the bark of *Morus nigra* [114].

The physical constants and spectroscopic properties (particularly NMR data) of both natural and synthetic ORV are presented in numerous reports [14,21,34,42,49,56,57,72,76,107,128]. The data for the crystal structure of ORV in the form of dihydrate are also available [129].

### 2.2. Qualitative and Quantitative Analysis

In recent decades, several analytical methods have been developed for detecting and quantifying ORV in plant materials. Most systems consist of two main parts: (*a*) separation, e.g., high-performance liquid chromatography (HPLC), and (*b*) detection, e.g., UV/photodiode array detection and/or high-resolution mass spectrometry (MS). For example, a reversed-phase HPLC system using a linear gradient of acetonitrile-phosphoric acid in water with UV detection was employed to determine the stilbenoids (including ORV) present in *Smilax china* [30]. The ORV contents in the fruits and leaves of eight species of *Morus* were analyzed by an HPLC system comprising a reversed-phase column with a methanol-water mixture and UV detection [108]. An LC-ESI-MS/MS (liquid chromatography/electrospray ionization–mass spectrometry) system was developed for the quantitation of oxyresveratrol content in the root of *Smilax china* [35,36].

Thin-layer chromatography (TLC)-based analytical methods have been suggested as a simpler alternative, requiring less sophisticated instrumentation than the HPLC. A validated TLC densitometric method for the determination of ORV contents in *Artocarpus lakoocha* heartwood and traditional drug ‘Puag-Haad’ samples was reported [46]. In another study, a TLC analytical system was developed and employed to detect ORV in *Morus alba* (root and bark) and *M. rubra* (stem and leaves), in comparison with the traditional HPLC method [98].

An analytical method based on a monoclonal antibody produced in mice was recently developed for the quantitation of ORV in plant materials and products [130]. The immunogen was generated from the Mannich reaction of ORV with cationized bovine serum albumin (cBSA). An antibody specific to ORV was obtained and then applied in the form of an indirect competitive enzyme-linked immunosorbent assay (ELISA) to determine the ORV contents in commercial products of *Artocapus lakoocha*.

## 3. Production

### 3.1. Chemical Synthesis

**Figure 3:**
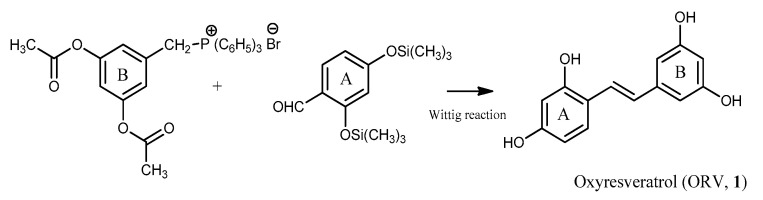
Synthetic route of ORV (**1**) through Wittig reaction.

The first chemical synthesis of ORV was reported in 1970 [131,132]. ORV was obtained by the Wittig reaction of triphenyl-[3,5-dihydroxybenzyl]-phosphonium bromide with silylated 2,4-dihydroxybenzaldehyde in the presence of an excess of phenyl lithium (Figure 3). This synthetic strategy was also used to prepare ORV and related compounds in a cytotoxicity study [133].

**Figure 4:**
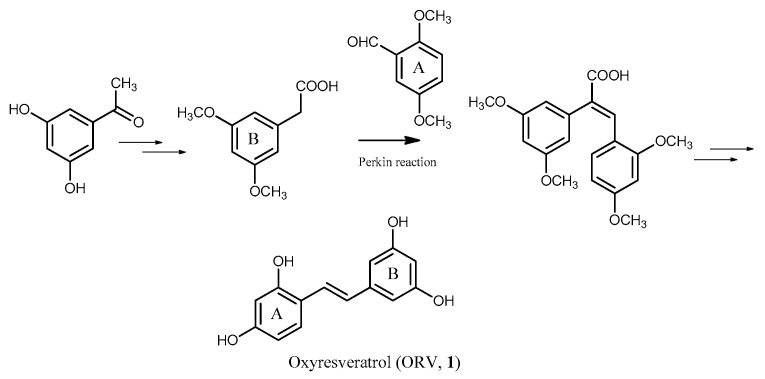
Synthetic route of ORV (**1**) through Perkin reaction.

The second synthetic route for the preparation of ORV and related analogs was based on the Perkin condensation reaction of 3,5-dimethoxyphenylacetic acid with a corresponding substituted phenylaldehyde [134,135]. First, 3,5-dimethoxyphenylacetic acid was prepared from 3,5-dihydroxyacetophenone through a methylation reaction and Willgerodt–Kindler rearrangement. For the preparation of ORV, the first step started with a Perkin reaction of 3,5-dimethoxyphenylacetic acid with 2,5-dimethoxyphenylaldehyde to provide 2,3-diarylcrylic acid with the two aromatic rings in a *cis*-relationship. This diarylcrylic acid intermediate was then decarboxylated with Cu/quinoline at 220 °C and then subjected to a simultaneous demethylation/isomerization process by AlI_3_ in CH_3_CN to furnish ORV (Figure 4).

**Figure 5:**
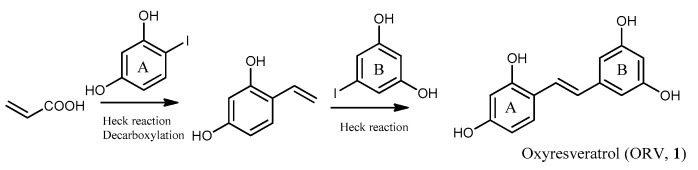
Synthetic route of ORV (**1**) through Heck reaction.

More recently, a simple two-step synthesis of ORV was reported [136]. 2,4-Dihydroxyiodobenzene was allowed to react with acrylic acid under Heck reaction conditions (Pd catalyzed reaction) to provide an intermediate that, after decarboxylation and coupling with 3,5-dihydroxyiodobenzene through Heck reaction, yielded ORV (Figure 5).

It is noteworthy that ORV is light-sensitive, as it was reported that this compound rapidly underwent a *trans*-*cis*-photoisomerization and photodegradation by UV irradiation in ethanol solution [137].

### 3.2. Biotransformation

#### 3.2.1. Enzymatic Deglycosylation

Glycosidic ORVs occur naturally in large amounts in several plants, and thus offer a promising source of starting materials for producing ORV through enzymatic deglycosylation. Several procedures for the removal of the sugar units have been reported. The first study describes the use of cellulase to catalyze the deglycosylation reaction of ORV glucosides in a *Veratrum patulum* extract [138]. ORV could also be produced from mulberroside A in a *Morus alba* root extract through a hydrolytic reaction catalyzed by the enzyme Pectinex [139,140], as well as by the enzyme extract obtained from the acid bacterium *Leuconostoc paramesenteroides* [141].

More recently, ORV was prepared from pinosylvin or resveratrol through a hydroxylation reaction catalyzed by unspecific peroxygenases from the basidiomycete fungi *Agrocybe aegerita* and *Coprinopsis cinerea* in the presence of H_2_O_2_ [142].

#### 3.2.2. Plant Cultures

An attempt to produce ORV from callus cultures of *Artocarpus lakoocha* was carried out, but only a trace amount of the target compound was detected [5]. By contrast, several protocols for producing ORV from *Morus alba* cultures were successfully established. In a *Morus alba* cell suspension, mulberroside A was generated and then deglycosylated by an endogenous enzyme to provide ORV in situ [143]. Large amounts of ORV could also be obtained from a *Morus alba* callus culture incubated with the elicitor 2-hydroxypropyl-β-cyclodextrin [144]. An immobilized cell culture of *Morus alba* that produced large amounts of muberroside A and ORV was also demonstrated [145]. The production of ORV could be increased by the addition of the precursor l-tyrosine or the elicitors methyl jasmonate and yeast extract. Similar enhancements were also observed when l-tyrosine or a combination of methyl jasmonate and yeast extract was added to the root culture of *Morus alba* [146].

## 4. Biological and Pharmacological Activities

### 4.1. Inhibition of Tyrosinase Melanogenesis and Browning

**Figure 6:**
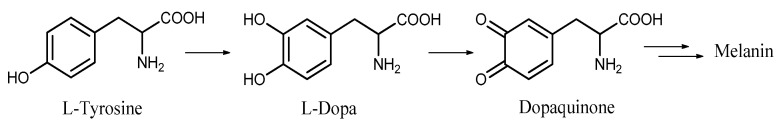
Functions of tyrosinase.

Tyrosinase is a copper-containing enzyme, and it is mostly known for its roles in the production of melanin pigments (melanogenesis) in skin, eyes, and hair, as well as in the browning of vegetables and fruits [147]. The enzyme catalyzes the conversion of l-tyrosine (monophenolase activity) or l-dopa (diphenolase activity) into dopaquinone which, upon cyclization followed by oxidation and polymerization, produces melanin (Figure 6).

Table 2 summarizes the studies on tyrosinase inhibitory activity and potential applications of ORV. It can be seen from the table that mushroom tyrosinase has been extensively used in the preliminary investigations.

ORV inhibited mushroom tyrosinase in a non-competitive manner when l-dopa was employed as the substrate [139,148,150]. This was also confirmed by a detailed study using combined techniques of fluorescence spectroscopy and circular dichroism (CD) [49]. However, when l-tyrosine was the substrate, differing results were obtained. The first study suggested a reversible noncompetitive mode of inhibition [47], but a subsequent report described ORV as a competitive inhibitor [77]. In more recent investigations, ORV was concluded to be a reversible mixed-type inhibitor, based on the *Ki* and *Ki*′ values obtained from the Lineweaver–Burk plots [139,155]. In addition, ORV was found to exhibit a synergistic inhibitory effect with dioscin, a steroidal saponin, in a study with mushroom tyrosinase [34].

Tyrosinase enzymes from other sources, such as murine (lysates of B16/B16-F10 cells) and human (lysates of HEK293-TYR or HEM cells), were also tested and found to be inhibited by ORV in a similar fashion [128,149]. Numerous studies of ORV were conducted on cellular tyrosinase. ORV showed suppression of melanogenesis in murine melanoma B16/B16-F10 cells [49,61,68,128,153,154,156,157,158] and human PIG1 melanocytes [152], all in a dose-dependent manner. ORV reduced tyrosinase-related protein-1 (TRP-1) and tyrosinase-related protein-2 (TRP-2) productions in B16-F10 cells via downregulation of microphthalmia-associated transcription factor (MITF) [68]. In a study on the binding of ORV to tyrosinase using fluorescence and circular dichroism spectroscopic techniques, it was found that there was a static interaction between ORV and the enzyme through van der Waals forces and hydrogen bonding [153]. Furthermore, the results from molecular modeling studies indicated that ORV did not bind directly to the copper ion of the enzyme but instead formed hydrogen bonds to nearby histidine residues (His259 and His263). These interactions help to explain the stability of the enzyme-inhibitor complex and the high inhibitory potency of ORV [97,153].

ORV was studied for effects on melanogenesis in several animal models. ORV suppressed melanogenesis in zebrafish embryos [97]. It reduced melanin production and simultaneously increased the life span of the nematode *Caenorhabditis elegans* [49]. Topical application of an extract of *Morus alba* (containing ORV) on the skin of brown guinea pigs attenuated UV-B-induced skin pigmentation [77]. In a similar study, the skin epidermal cells of animals were collected and examined by Western blot and qRT-PCR mRNA analysis. The results indicated that ORV reduced the levels of tyrosinase, TRP-1, and MITF by suppressing the expression of Tyr, Trp1, and Mitf genes [159].

When evaluated on human skin equivalents, MelanoDerm (MEL-300-B), ORV showed a profound hypopigmentation effect [60]. In human trials, an *Artocarpus lakoocha* extract (ALE), which contained ORV about 80% *w*/*w*, was studied for skin whitening effects. A 0.10% ALE lotion was applied onto the cheeks or upper arms of the volunteers, two times daily for four weeks. Significant whitening effects were observed after two weeks of application and continued toward the end of the study [160]. ORV appears safe for external use as it did not cause any irritation, edema, or erythema in an assessment using a model of white guinea pigs [161].

The potential application of ORV in the agricultural area was also explored. The optimal anti-browning effect of ORV in cloudy apple juices was attained at a concentration of 0.01%. Fresh-cut apple slices required some additives, such as isoascorbic acid, calcium chloride, and acetylcysteine, to achieve similar effects [78].

### 4.2. Antioxidant and Anti-Inflammatory Activities

ORV has been extensively investigated for antioxidant and anti-inflammatory activities as these attributes are widely considered to be the underlying mechanisms of several pharmacological properties. There is a large body of literature in this area, as illustrated in Table 3.

ORV has been studied for inhibitory activity against several types of reactive chemical species, such as DPPH, O_2_^• −^, HO^•^, ABTS^• +^, H_2_O_2_, and NO^•^ (Table 3). The protective activity of ORV against lipid peroxidation has been investigated in several testing systems, including FeSO_4_/H_2_O_2_ in rat liver microsomes [106], FeSO_4_/ascorbic acid in rat brain homogenates [76], and AAPH (2,2′-Azobis-2-methyl-propanimidamide dihydrochloride) in liposomes [54]. ORV showed weak inhibitory activity on COX-1, COX-2, and lipoxygenase-1 [42,166], and toward DMBA (7,12-dimethylbenz [*a*] anthracene)-induced preneoplastic lesions with MMOC (mouse mammary in organ culture) [42]. ORV inhibited the platelet-activating factor (PAF)-induced release of β-glucuronidase in rat polymorphonuclear leukocytes [117].

Many studies aimed to investigate the connections between the antioxidant/anti-inflammatory activity and the neuroprotective effects of ORV. ORV was found to protect cultured rat cortical neurons from *N*-methyl-D-aspartate (NMDA)-induced toxicity by suppressing both the generation of ROS and the increase in intracellular Ca^2+^ concentration [31]. In a study using murine N9 microglial cells and primary mixed glial cultures, ORV showed strong NO scavenging activity but did not inhibit the activity of iNOS (inducible nitric oxide synthase) and the expression of iNOS protein [94]. In a study with cultured P19 cells under oxidative stress induced by serum deprivation, ORV prevented neuronal cell death and improved neurite growth [167]. In murine BV-2 microglial cells treated with lipopolysaccharide (LPS), ORV suppressed the release of NO, TNF-α, iNOS, IL-1β, and IL-6 through MAPKs (ERK1/2, JNK, and p38) and NF-κB signaling pathways [168]. ORV showed protective activity against H_2_O_2_-induced injury in PC12 cells in a dose-dependent manner [172].

Murine macrophage RAW 264.7 cells have been used in several studies of anti-inflammatory activity. ORV reduced lipopolysaccharide (LPS)-induced NO production by suppressing iNOS expression, with very little effect on iNOS activity [106,169]. ORV also decreased prostaglandin E2 (PGE2) generation through the downregulation of COX-2 protein expression [47,169]. Besides, ORV inhibited the production of interleukin 6 (IL-6) and granulocyte macrophage colony-stimulating factor (GM-CSF), as well as the phosphorylation of mitogen-activated protein kinases (MAPKs), extracellular signal-regulated kinase (ERK), and c-JunN-terminal kinase (JNK) [170].

Some studies on the anti-inflammatory activity of ORV were carried out using human cell lines. In human periodontal ligament (hPDL) cells treated with LPS (lipopolysaccharide), ORV inhibited the expression of IL-6 and IL-8 at both mRNA and protein levels [173]. In a study with human primary epidermal keratinocytes irradiated with UV-A, ORV prevented the cell damage by suppressing the generation of ROS and nitrotyrosine and the increase in the levels of 8-hydroxy-2′-deoxyguanosine (8-OHdG) and cyclobutane pyrimidine dimers (CPDs) [68]. ORV protected human lens epithelial cells (HLECs) from H_2_O_2_-induced oxidative stress and inhibited apoptosis through the activation of the Akt and heme oxygenase-1 (HO-1) pathways [174]. FOXO3a (Forkhead box O3) is a transcription factor known to be important in several biological functions, such as DNA repair and detoxification of ROS. ORV was shown to suppress the ROS level in human embryonic fibroblast (HEF) cells by upregulating Manganese superoxide dismutase (MnSOD) through the activation of FOXO3a [175].

ORV suppressed the migration of human Jurkat leukemic T cells induced by SDF-1 (stromal cell-derived factor 1) via inhibition of the MEK/ERK pathways [93]. In a study with interleukin-1β (IL-1β)-stimulated C28/I2 human chondrocyte cells, ORV suppressed MMP-13 (matrix metalloproteinase-13) production [169]. In HMC3 human microglial cells treated with IL-1β, ORV decreased the release of IL-6 and MCP-1 through the suppression of the PI3K/AKT/p70S6K and ERK1/2 MAPK pathways [181].

In a study with MCF-7 (human breast adenocarcinoma) cells, ORV was found to induce cell proliferation by activating the estrogen receptor (ER)-mediated transcription and increase the levels of estrogen-targeted gene expression and ERα/β protein production [176]. The molecular docking scores indicated that the compound could bind to and act as an agonist of both ER receptors. A detailed study in LPS-stimulated RAW264.7 cells showed that ORV downregulated NF-κB transcription and suppressed the increase in phospho-IκB-α and phospho-p65 protein levels in an ER-dependent manner [176]. These experimental data suggested that estrogenic receptor signaling is one of the possible mechanisms of action. In a recent molecular modeling study aiming to investigate the potential of ORV to treat pelvic inflammatory disease, ORV showed a high binding affinity to matrix metalloprotein-9 [185].

ORV showed significant anti-inflammatory activity in a rat model of carrageenan-induced hind-paw edema (CARA) [106]. Subsequent studies with LPS-simulated RAW 264.7 cells suggested that the key mechanisms involved the suppression of the nuclear translocation of NF-κB, the production of prostaglandin E2 (PGE_2_), and the activity of cyclooxygenase-2 (COX-2) [106,169].

In rat mast cells, ORV inhibited histamine release induced by concanvalin A [122]. In a study using a mouse model of allergic airway inflammation, the animals were immunized by intraperitoneal injection with OVA. Treatment with ORV via the intraperitoneal route reduced the total leucocyte counts in both the blood and the bronchoalveolar lavage fluid. The molecular mechanisms involved the attenuation of inflammatory cell infiltration and goblet cell hyperplasia, as well as the suppression of Th2 (T helper cells)-type immune response [182].

The demonstrated anti-inflammatory properties of ORV have also pointed to the potential of ORV as a therapeutic agent for inflammatory bowel diseases, such as Crohn’s disease and ulcerative colitis. In human intestinal Caco-2 cells, ORV showed a strengthening effect on tight junctions [177,178]. Studies in human intestinal goblet LS 174T cells revealed that ORV could stimulate mucin production, and the activity was found to be NAD+-dependent and involved the induction of autophagy through the endoplasmic reticulum (ER) stress pathway [179,180]. An extract of *Morus alba* containing oxyresveratrol was studied for therapeutic potential in colitis using the models of LPS-treated RAW 264.7 murine macrophage cells and dextran sodium sulfate (DSS)-treated mice. The extract suppressed inflammation and stimulated mucin production in a manner similar to that of ORV [171]. In a rat model of dextran sulfate sodium (DSS)-induced colitis, ORV reduced inflammation within the intestine by inhibiting COX-2, iNOS expression, and apoptosis of the epithelial cells [184]. Another investigation using a mouse model of ethanol-induced ulcer found that pretreatment with oral ORV reduced ulcer scores and ulcer index, as determined from the increased gastric pH and the decreased expression levels of IL-6, TNF-α, NF-ĸB, and COX-2, with unaltered COX-1 expression levels [183].

It should be mentioned that, despite numerous reports on its antioxidant activity, as well as its inhibitory activity against tyrosinase (a multi-copper-containing enzyme), ORV was reported to behave like a pro-oxidant in the presence of Cu^2+^ ions in a recent study [186].

### 4.3. Neuroprotective Activity

#### 4.3.1. Ischemia and Stroke

An extract prepared from *Smilacis china* rhizome (with ORV as the major component) was studied for potential applications in neurological disorders in a rat model of MCAO-induced focal cerebral ischemia. The orally administered extract reduced the infarction volume with no significant change in body temperature [31]. In another investigation, a transient rat middle cerebral artery occlusion (MCAO) model was employed. Intraperitoneally administered ORV reduced the brain infarct volume, cytochrome c release, and caspase-3 activation, resulting in a decrease in the number of apoptotic neurons [187]. The high levels of ORV observed in the infarct region suggested a high blood–brain barrier (BBB) permeability of ORV in the bloodstream. The direct action of ORV on the affected area implied its potential use in stroke [188]. Another study revealed that ORV could also inhibit neuronal cell death induced by stretch injury, implying an application in traumatic brain injury [189].

#### 4.3.2. Alzheimer’s Disease (AD)

ORV inhibited butyryl cholinesterase with a potency close to that of galantamine, a cholinesterase inhibitor drug used for treating AD [44]. It also blocked a recombinant human β-secretase (β-site amyloid precursor protein cleaving enzyme 1, BACE1) in a non-competitive fashion, with a *Ki* value of 5.4 μM, as determined from the Dixon plot [28]. However, in a recent virtual evaluation using the 3D BACE1 structure retrieved from the protein data bank (PDB), ORV did not show a high docking score [190]. In a study using cultured rat cortical neurons treated with amyloid β protein (25–35) (Aβ 25–35), ORV prevented cell death by suppressing the levels of cytoplasmic [Ca^2+^] and reducing glutamate release and ROS generation [27]. In Neuro2a neuroblastoma cells, ORV induced caspase-3-dependent cell death and autophagy, and inhibited γ-secretase, one of the key enzymes involved in the production of β-amyloid (Aβ) from the amyloid precursor protein (APP) [191]. ORV reduced the secretion of soluble APPs in Neuro2a cells and decreased the level of Aβ1–42 in HEK293 human embryonic kidney cells [191]. In a study with primary cortical astrocytes, ORV promoted autophagy through the activation of AMP-activated protein kinase and unc-51-like autophagy activating kinase 1 (ULK1). In addition, ORV attenuated corticosterone-induced APP production in these neuronal cells [192].

In an AD model of mice injected intracerebroventricularly with the amyloid β peptide Aβ25–35, oral ORV (in a self-microemulsifying drug delivery system, SMEDDS) reduced behavior impairments, lipid oxidations, and neuronal loss in the hippocampus [193].

#### 4.3.3. Parkinson’s Disease (PD)

In an in vitro PD model of SH-SY5Y cells treated with 6-hydroxydopamine (6-OHDA), ORV attenuated the increase in lactate dehydrogenase level, caspase-3 activity, and ROS production induced by 6-OHDA. ORV exerted activity through suppression of the c-Jun N-terminal kinase (JNK) pathway and the elevation of SIRT1 protein [79]. It should be noted that the cytotoxic potential of ORV against SH-SY5Y cells has been controversial. In an investigation, ORV was reported to be cytotoxic toward SH-SY5Y cells at as low as 120 μM [194]. However, a more recent study reported that ORV did not show toxicity at a concentration up to 200 μM in a 24 h incubation [195]. Moreover, in hybrid murine neuroblastoma-glioma Mes23.5 cells treated with 6-OHDA, ORV prevented cell death by inhibiting the transcription of activating transcription factor 4 [196].

In an in vivo PD model of rats injected with 6-OHDA at the median forebrain bundle, ORV given by oral gavage could reduce motor impairments, as examined by rotation, cylinder, and rotarod tests, but the compound did not prevent dopaminergic neuronal loss [197]. In rats subcutaneously injected with rotenone, oral administration of ORV reduced motor impairments and MDA levels, enhanced catalase activity, and preserved dopaminergic neurons in the substantia nigra [198].

#### 4.3.4. Other Models of Neuroprotective Activity

Oral administration of ORV to mice injected intraperitoneally with ethanol showed a decrease in DNA damage in the cerebellum and cerebral cortex [41]. In mice treated with an intracerebroventricular injection of kainic acid (KA), pretreatment with oral ORV prevented hippocampal neuronal cell death through the suppression of Forkhead homeobox type O3a (FoxO3a) levels and pFoxO3a protein expression in the hippocampus [199].

ORV was investigated in a rat model of spinal cord injury (SCI). Intraperitoneal injection of ORV improved motor impairments and attenuated an increase in spinal cord water content. Biochemical and Western blot analyses of the blood obtained from the eye socket and spinal cord tissue of the animals revealed that ORV suppressed the activities of NF-κB/p65, TNF-α, IL-1β, and IL-6, and reversed MDA, SOD, GSH, and GSH-PX levels through the iNOS, COX-2, and GM-CSF/Nrf2 pathways [200].

### 4.4. Hepatoprotective Activity

Human liver Hep G2 cells have been used in several studies. ORV was reported to protect Hep G2 cells from tacrine-induced toxicity [75]. In HepG2 cells treated with nicotine, ORV prevented cytotoxicity by reducing ROS generation in the mitochondria and upregulating CYP2A6 expression, thereby accelerating the conversion of nicotine into cotinine [35]. ORV could also reduce ROS production and cell death triggered by *tert*-butyl hydroperoxide [201]. In a study of the effects of ORV on microsomal enzymes, ORV showed moderate inhibition of CYP3A4/5 but weak activity on CYP1A2 and CYP2C8 [202].

ORV was also evaluated for potential applications in nonalcoholic fatty liver disease (NAFLD). The compound inhibited hepatic lipogenesis in Hep G2 cells by suppressing LXRα–SREBP-1c-mediated lipogenic gene induction through the LKB1/AMPK pathway [203]. In mice fed with a high-fat diet (HFD), the fat accumulation was attenuated by oral administration with ORV without bodyweight reduction. ORV reduced lipid droplets and hepatic steatosis regions, as well as several serum parameters, including plasma fasting glucose, total cholesterol, and LDL levels [203]. ORV showed protective activity on the liver of mice fed with ethanol (EtOH) [16]. Oral administration of ORV restored the decreased levels of reduced glutathione and antioxidant enzyme activities induced by EtOH. ORV also suppressed the elevated levels of lipid peroxidation, cytochrome P450 2E1 activity, iron concentration, and mitochondrial permeability.

In mice injected intraperitoneally with carbon tetrachloride (CCl_4_), oral administration of ORV prevented acute liver injury through the suppression of inflammatory cell infiltration and increases in the levels of alanine transaminase (ALT) and aspartate aminotransferase (AST) [201]. The hepatoprotective activity of ORV was also confirmed by a study in rats treated with CCl_4_ [204]. Intragastrical administration of ORV ameliorated liver oxidative damage. Detailed studies revealed that ORV reduced the mRNA transcription expressions of α-smooth muscle actin (α-SMA), desmin, and two matrix metalloproteinases (MMP2 and MMP9). ORV also caused the downregulation of transforming growth factor β1 (TGF-β1), p-small mother against the decapentaplegic protein (Smad) l/2, and p-extracellular signal-regulated kinases (ERK) l/2 in the liver tissue. In another study, ORV was also found to inhibit the activation of Smad 3 [205]. When compared to resveratrol (**2**), ORV (**1**) showed a higher protective effect. It was proposed that the OH group at C-2 of **1** provides additional electron delocalization, resulting in higher antioxidative and hepatoprotective activities [204].

ORV also prevented liver damage in mice injected with alloxan intraperitoneally. Oral ORV suppressed the alloxan-induced elevation of ALT and AST activities and the generation of malondialdehyde. ORV also increased the levels of reduced glutathione concentration, as well as superoxide dismutase and catalase activities [81]. Similar effects on the activities of these two enzymes were observed when ORV was given to mice under isoniazid-induced oxidative stress [206].

In mice injected with lipopolysaccharide and D-galactosamine (LPS/D-GalN), acute liver injury could be prevented by oral administration of ORV. The compound decreased the activities of ALT and AST and suppressed the levels of enzymes and products caused by oxidative stress, as well as the expressions of several inflammatory mediators. The mechanisms involved the downregulation of the Toll-like receptor 4/nuclear factor-kappa B (TLR4/NF-κB) signaling and the activation of the Kelch-like ECH-associated protein 1(Keap1)-nuclear factor erythroid 2-related factor 2 (Nrf2) pathway [207,208].

### 4.5. Anticancer Activity

Although a few reports on the in vitro cytotoxicity of ORV against several cancer cell lines have been published, so far, no studies in animals have been reported. In primary screening, ORV showed weak cytotoxicity against murine leukemia P-388 cells [110], as well as several human cancer cell lines, including KB, BC, NCI-H187, HepG2, A549, Col2, A2780, BGC-823, and MCF-7 [117,133,150,209]. However, full *O*-methylation of ORV led to a substantial increase in cytotoxicity [150]. This phenomenon was also observed for tetra-*O*-methyl ORV, which suppressed the viability and migration of human MCF-7 breast tumor cells [210].

Interestingly, ORV induced caspase-independent cell death in the highly chemo-resistant, triple-negative human breast cancer MDA-MB-231 cell line through the generation of ROS [211]. In a study with MCF-10A cells, ORV weakly inhibited NF-κB/DNA binding induced by the carcinogen TPA (12-O-tetradecanoylphorbol-13-acetate) [212]. ORV also showed selective cytotoxicity against human gastric cancer BGC-823 cells [117].

In osteosarcoma cell Saos-2 cells, ORV inhibited cell viability and induced apoptosis by suppressing the activation of STAT3 (signal transducer and activator of transcription 3) [213]. ORV also induced apoptosis of human bladder cancer T24 cells in a dose- and time-dependent manner [214]. In a study to evaluate the antiproliferative and antiangiogenic effects on HSC-3, HN-8, and HN-30 squamous cell carcinoma cells, ORV showed a dose-dependent inhibition of cell growth through the suppression of vascular endothelial growth factor (VEGF) expression at both the mRNA and protein levels [215]. ORV suppressed H22 hepatocellular carcinoma growth and lymph node metastasis [216].

A few virtual screening studies focusing on the anticancer activity of ORV have been reported. In a molecular docking study, ORV showed weak affinity with human cyclophilin D (CyPD) protein, the target for the treatment of oral carcinoma [217]. Using multi-spectroscopic techniques and molecular docking analysis, combined with in vitro data, a study predicted that ORV could interact with human serum albumin (HSA) and enhance the anticancer activity of the drug mitoxantrone [218]. Through bioinformatic analysis of the biochemical data, it was proposed that ORV could exert its cytotoxic potential against HCT116 and SW620 human colon cancer cells by inducing apoptosis and suppressing cell proliferation [219].

### 4.6. Metabolic Disorders

#### 4.6.1. Glucose Metabolism

α-Glucosidase is an intestinal enzyme responsible for digesting dietary carbohydrates into glucose for intestinal absorption. Inhibitors of this enzyme have been used clinically to control postprandial hyperglycemia. In a study with α-glucosidase from *Saccharomyces cerevisiae*, ORV inhibited the enzyme in a non-competitive manner, with a higher potency than that of the drug acarbose [220]. The ORV–enzyme complex was studied by analysis of the fluorescence quenching spectrum and confirmed by a molecular docking study. However, when evaluated in differentiated Caco-2 cells, ORV showed weaker inhibitory activity than acarbose on maltose hydrolysis [221]. More importantly, ORV did not inhibit pancreatic amylase. This should be an advantage of ORV, as ORV should not cause abdominal bloating, flatulence, or diarrhea, as seen in the drug acarbose. ORV also reduced the glucose transport in Caco-2 cells in a dose-dependent manner, and this property might enhance its ability to suppress postprandial hyperglycemia [222].

Glucokinase is also an enzyme that has an important role in blood glucose homeostasis, as it controls glucose disposal and glycogen synthesis in the liver. The results from an in vitro assay in HepG2 cells, together with the data from a molecular docking study, suggested that ORV could bind to glucokinase and stimulate the activity of the enzyme [223].

ORV showed strong antiglycation activity. It blocked the formation of advanced glycation end products (AGEs) more effectively than the positive control aminoguanidine [54,224,225].

The postprandial hypoglycemic effects of ORV and *Morus alba* extracts were investigated in streptozotocin-induced diabetic rats [85] and mice [96]. Oral administration of ORV reduced the fasting plasma glucose level but increased the hepatic glucose transporter 2 transcription and glycogen content, without effects on plasma insulin concentration and intestinal disaccharidase activity [96].

ORV weakly stimulated insulin secretion in MIN 6 (mouse insulinoma 6) cells under H_2_O_2_-induced oxidative stress [226]. In insulin-resistant C57BLKS/J db/db mice fed with a high-fat diet, oral ORV suppressed the fasting blood glucose level, elevated the levels of insulin and C-peptide, and improved pancreatic β-cell dysfunction [227]. ORV stimulated insulin secretion in INS-1 cells and showed an anti-glycation potential through the inhibition of advanced glycation end-product formation [227].

#### 4.6.2. Lipid Metabolism

ORV was investigated for effects on adipogenesis in 3T3-L1 cells. The compound decreased cellular triacylglycerol accumulation by inducing cell cycle arrest in the G1 phase through the downregulation of cyclin D1, cyclin-dependent kinase 4 (CDK4), and cyclin-dependent kinase inhibitor 1B (P27/kip1) expression [228]. A study with a *Morus alba* extract containing ORV as the major component gave comparable results [90]. In a recent in silico study, ORV showed a high affinity to PPAR γ, implying a promising ADME profile [229]. ORV showed significant inhibitory activity on rat 3-hydroxy-3-methylglutaryl coenzyme A (HMG-CoA) reductase and squalene synthase [138]. A study in C2C12 myoblasts revealed that ORV could activate the expression of estrogen receptor-related receptor (ERR)/PGC-1β complex-mediated gene and, thus, might have the ability to increase energy expenditure [230].

In a study with Triton WR-1339-induced hyperlipidemic rats, pretreatment with oral ORV reduced the levels of serum lipids but increased high-density lipoprotein cholesterol (HDL-C) levels [231]. In high-cholesterol diet (HCD)-induced hyperlipidemic rats, oral ORV showed a decrease in serum lipids, coronary artery risk index (CRI), and atherogenic index (AI), but not HDL-C [231]. Comparable results were obtained for a *Morus alba* extract containing high amounts of ORV [232]. The anti-hyperlipidemic effects were in parallel with the elevated expressions of lecithin cholesterol acyltransferase (LCAT), cholesterol 7α- hydroxylase (CYP7A1) and low-density lipoprotein receptor (LDLR), and the suppression of 3-hydroxy-3-methylglutaryl coenzyme A reductase (HMGCR) and acyl coenzyme A cholesterol acyltransferase-2 (ACAT2) levels [232]. The anti-obesity activity of ORV was also observed in a study with high-fat diet (HFD)-fed mice [233]. ORV was found to attenuate lipid accumulation and adipocyte differentiation in 3T3-L1 and C3H10T1/2 cells, and to suppress an increase in energy expenditure in C3H10T1/2 adipocytes through Foxo3a-mediated induction of the thermogenic genes [233].

Several studies were carried out on C57BL/6 obese mice fed with a high-fat diet (HFD) [234,235,236]. Supplementation with ORV reduced insulin resistance, hyperglycemia, and hepatic steatosis. Several biochemical cascades were involved in the observed hypolipidemic effects [234,235].

### 4.7. Antimicrobial Activity

#### 4.7.1. Antiviral Activity

ORV weakly inhibited the growth of human immunodeficiency virus type 1 (HIV-1) in vitro [37,52]. The compound exhibited moderate inhibitory activity in vitro against herpes simplex types 1 and 2 (HSV-1 and HSV-2) in an inactivation assay [52]. A subsequent study revealed that ORV inhibited HSV-1 growth at the early and late phase of replication, suppressed late protein synthesis, and possessed in vitro synergistic effects with the drug acyclovir [237]. In an in vivo study with mice cutaneously infected with HSV-1, oral administration of ORV delayed herpetic skin lesion development. Topical application of ORV in the form of an ointment or a cream significantly slowed the development of skin lesions and rescued the mice from death [237,238], but less efficacy was observed for ORV in the form of microemulsion [239].

ORV exhibited moderate inhibitory activity in vitro against other viruses, including varicella-zoster virus (VZV or chickenpox virus) [240] and African swine fever virus (ASFV, a porcine pathogen) [241], but no activity was observable in chikungunya virus (CHIKV) [242].

#### 4.7.2. Antibacterial Activity

ORV showed recognizable inhibitory activity in vitro against the periodontal pathogenic bacteria *Porphyromonas gingivalis* and *Aggregatibacter actinomycetemcomitans* [173]. In a study with *Streptococcus mutans*, a pathogen of dental caries, ORV inhibited bacterial growth, biofilm formation, and acid production in a dose-dependent manner [243].

A preliminary study on the antibacterial activity of ORV against *Staphylococcus aureus*, *Bacillus subtilis*, *Micrococcus flavus*, *Streptococcus faecalis*, *Salmonella abony*, and *Pseudomonas aeruginosa* was described [114]. ORV showed weak antibacterial activity in vitro against six standard strains and two clinical isolates of *Staphylococcus aureus* [244]. When evaluated against methicillin-resistant *Staphylococcus aureus* (MRSA), ORV displayed discernable inhibitory activity and exerted synergistic effects with the drugs ciprofloxacin and gentamicin by increasing the permeability of bacterial cell membrane and inhibiting the enzyme ATPase [86,245]. ORV inhibited the growth of the uropathogenic *Escherichia coli* by suppressing biofilm formation, swarming motility, fimbriae production, and hemagglutination ability of the bacteria [246].

The effect of ORV on quorum sensing (QS), a Gram-negative bacterial cell-to-cell communication system, was studied in *Chromobacterium violaceum* CV026 [247]. ORV inhibited the bacterial signaling, as indicated by the reduced amounts of the pigment violacein. Further studies in *Pseudomonas aeruginosa* PAO1 revealed that ORV reduced the production of pyocyanin and swarming motility, without effects on bacterial growth [247]. In a study of the effects of ORV on the composition of fecal microbiota grown under anerobic conditions, ORV decreased the Firmicutes to Bacteroidetes ratio and the relative abundance of strains from the genus *Clostridium* but increased the growth of *Faecalibacterium prausnitzii*. As a result, ORV was reduced to dihydrooxyresveratrol by hydrogenation at the olefinic bond [248,249].

#### 4.7.3. Antifungal Activity

ORV, as a phytoalexin, has inspired a great deal of interest in its antifungal activity [11,71,73,87,125,250,251,252]. Studies on the heartwood of the osage orange *Maclura pomifera* (originally described as *Toxylon pomiferum*) suggested that large amounts of ORV were produced in response to the threat from the wood-decaying fungi *Trametes versicolor* and *Gloeophyllwn trabeum* [71,73]. A recent investigation revealed that ORV inhibited glutathione transferase Omega (TvGSTO2S), a wood degrading enzyme found in *Trametes versicolor* [251].

The antifungal effects of ORV on several dermatophytes, including *Trichophyton rubrum*, *Trichophyton mentagrophytes*, *Trichophyton tonsurans*, *Microsporum canis*, *Microsporum gypseum*, and *Epidermophyton floccosum*, were reported [125,250]. ORV also showed synergistic effects with the antifungal drug miconazole nitrate against *T. rubrum* [87]. The inhibitory activity of ORV on *Candida albicans* is still controversial. In previous studies [125,250], ORV showed no activity against *Candida albicans* but, in a more recent investigation [252], ORV was reported to inhibit the growth of *Candida albicans* by binding to the DNA, causing DNA cleavage and apoptotic cell death.

### 4.8. Other Activities

ORV showed weak insecticidal activity against the Colorado potato beetle *Leptinotarsa decemlineata*, as compared with the pesticide lambda-cyhalothrin [10]. ORV also exhibited weak larvicidal activity against the mosquito *Aedes aegypti*, as compared with permethrin [40]. ORV weakly inhibited the promastigotes and cellular amastigotes of *Leishmania amazonensis*, the parasite that causes cutaneous leishmaniasis in humans [253]. ORV was reported to show modest antiplasmodial activity against the chloroquine-resistant *Plasmodium falciparum* 3D7 and Swiss mice infected with *Plasmodium berghei* [43].

ORV demonstrated inhibitory activity on several enzymes, including rat brain monoamine oxidase A [107], rat adenosine triphosphatase (ATPase) [254], human cytochrome P450s (CYPs) 1A1, 1A2, and 1B1 [255], xanthine oxidase [33,256], phosphodiesterase-4 (PDE4) [257], and trypsin and lysozyme [258].

A study using UV and Raman spectroscopic techniques indicated that the toxicity of the carcinogen crotonaldehyde could be inhibited by ORV [259]. ORV showed muscle relaxant activity on rat tracheal smooth muscles, both in normal conditions and on contractions induced by histamine [260].

The anti-atherosclerotic and anti-fibrotic potentials of ORV were studied in a model of atherosclerosis-prone apolipoprotein E-deficient (Apoe^−/−^) mice fed with a high-fat diet. ORV reduced myocardial fibrosis development by suppressing the fibrotic and atherosclerotic markers/molecular signals in the CD137/Smad/NFATc1 pathway through the upregulation of miR-145 [261].

ORV expanded the life span of *Caenorhabditis elegans* (*C. elegans*) by increasing the mRNA expression of sir-2.1 and aak-2 genes and the expression level of SIR-2.1 protein and by activating the AMP-activated protein kinase (AMPK) pathway [262]. Higher lifespan extension could also be obtained with ORV complexed with a hyper-branched cyclodextrin-based nanosponge [263]. In *Saccharomyces cerevisiae*, ORV increased the lifespan of yeast via inhibition of the enzyme phosphodiesterase type 2 (PDE2) [264]. Anthelmintic activity against *Fasciola gigantica* was reported for an extract of *Artocarpus lakoocha* (no amount of ORV was described) [265].

### 4.9. Comparative Activities of Oxyresveratrol (***1***) and Resveratrol (***2***)

Resveratrol (**2**) is one of the most studied natural stilbenes due to its wide range of promising health benefits [266]. As mentioned earlier, ORV (**1**) is believed to be biosynthetically derived from (**2**), and several comparative studies of the biological/pharmacological activities of the two stilbenes have been reported. Table 4 highlights selected studies.

Although generalization of these activities is not possible, we can make some observations. For example, ORV (**1**) possesses higher antioxidant activity than resveratrol (**2**) in all the test models, except in the ORAC assay. This is because the antioxidant mechanism in the ORAC assay is based on a hydrogen atom transfer (HAT) [267]. The other evaluation methods involve a single electron transfer (SET), a mixed HAT/SET, or a reaction adduct formation mechanism [268,269], all of which benefit from the additional electron delocalization from the OH-2 group in (**1**). This potent antioxidant activity may also be the underlying mechanism for the neuro- and hepato-protective activities of (**1**) [79,196,197,199,204]. In a recent molecular docking study, this OH-2 was found to form a hydrogen bond with the Met280 residue of the enzyme tyrosinase [153]. This interaction might also be the key element that accounts for the superior inhibitory activity of (**1**).

## 5. Pharmacokinetic Profile

The first study concerning the metabolism of ORV was carried out on a *Morus alba* extract containing ORV 3′,4-diglucoside (**3**) (also known as mulberroside A) as the major component. Experimental evidence suggested that, after oral administration of the extract to the rats, mulberroside A was absorbed and metabolized in the liver, released back into the bloodstream, and then excreted in the bile and urine in the form of ORV, ORV 2,3′-*O*-β-D-diglucuronide, and ORV 2-*O*-β-D-glucuronide-3′-*O*-sulfate [270,271]. However, in a more recent study, the results from analysis of the intestinal contents and feces of the animals indicated that hydrolysis of mulberroside A occurred before intestinal absorption [272].

The results from analysis of the plasma collected from rats orally administered with ORV indicated that ORV was rapidly absorbed from the gastrointestinal tract (*t*_max_ 15 min) and excreted in the bile and urine [273,274,275]. Further studies on the rat urine and bile samples by HPLC-MS/MS revealed that monoglucuronide and monosulfate of ORV were the major metabolites [276,277]. Several studies concluded that oral ORV was rapidly absorbed, and ORV in solution was absorbed and eliminated faster than in the form of suspension [278,279,280].

Recently, the metabolism of ORV in humans was determined, based on the evidence obtained from the studies on (*a*) the breakdown of mulberroside A by human intestinal bacteria and (*b*) the transport of mulberroside A and ORV across Caco-2 cell monolayers [281]. In humans, mulberroside A is rapidly deglycosylated by intestinal bacteria to provide two monoglucosides and ORV sequentially. ORV is then quickly absorbed and undergoes hepatic conjugation to provide ORV 2-*O*-β-D-glucuronide and ORV sulfate. A further study using human liver and intestinal microsomes revealed that 2-*O*-β-D-glucuronide is the major metabolite, and UDP-glucuronosyltransferases (UGTs) are the enzymes involved in the transformation [282].

A study of the effect of piperine on the pharmacokinetic profile of oral ORV was conducted [283]. The oral availability of ORV was enhanced twofold when given in a combination with piperine.

## 6. Delivery Systems

At present, there are only a few reports on the pharmacological activities of ORV. In vivo experiments are difficult to design because ORV has a low water solubility, poor bioavailability, and low stability compared with other bioactive candidates. In the last decade, several approaches to overcome these hurdles have been reported.

Encapsulation with cyclodextrins (CDs) is a promising strategy to improve the stability and oral availability of the guest molecule. Several preliminary studies showed that ORV could form a complex with several types of CDs, such as β-cyclodextrin (β-CD), hydroxypropyl-cyclodextrin (HP-β-CD), and methyl-β-cyclodextrin (M-β-CD), with varying *K_F_* values depending on several factors, such as temperature and pH [284,285,286,287]. The optimal formulations of ORV-CD complex powder could increase the water solubility of ORV up to 100-fold with enhanced stability and an unaltered dissolution rate [288]. A juice or milk product fortified with an ORV/β-CD complex was found to be stable for 5 weeks in typical storage, and was still bio-accessible, with a higher bacteriostatic effect, than free ORV [289]. In a recent development, ORV/CD-nanosponge complexes showed higher resistance to in vitro degradation and a higher inhibitory activity against prostate (PC-3), colon (HT-29 and HCT-116), and prostate (DU-145) cancer cells, as compared with free ORV [290,291].

A study compared two lipid-based systems for oral delivery of ORV, i.e., nanostructured lipid carriers (NLC) and solid lipid nanoparticles (SLN) in terms of physicochemical properties and oral availability [292]. The optimized ORV-NLC formulation showed preferable characteristics compared to the ORV-SLN, including smaller nanoparticle sizes with homogeneous size distribution and higher zeta potential, a higher percentage of entrapment, and better stability. It was concluded that the ORV-NLC showed a more sustained release and improved bioavailability of ORV.

In another study, a self-microemulsifying drug delivery system (SMEDDS) for oral delivery of ORV was developed [293]. The ORV-SMEDDS showed more favorable physical properties than both the ORV-NLC and the ORV-SLN, with higher drug loading, smaller particle sizes, and narrower size distribution [294]. All three delivery systems showed enhanced permeability and reduced efflux transport across the Caco-2 monolayer compared to the unformulated ORV. The ORV-SMEDDS showed slightly higher toxicity on the Caco-2 cells than the ORV-SLN and ORV-NLC. The ORV-SMEDDS formulation, however, was successfully employed for the study of a rat model of AD [193]. In the latest developments, several nanoemulsions of ORV, with improved oral bioavailability and transdermal absorption, have been reported [295].

As mentioned earlier, a novel approach to improve the pharmacokinetic profile of ORV was studied in rats using piperine as the bioenhancer. The oral bioavailability of ORV was increased twofold when ORV was co-administered with piperine [283].

Several attempts were also reported to improve the water solubility, stability, and bioavailability of ORV by crystallization with different organic molecules. Cocrystals of ORV and citric acid (CTA) were obtained using the ethyl acetate-assisted grinding method. The ORV-CTA cocrystals showed increased water solubility and enhanced permeability of ORV across the Caco-2 monolayer, without toxicity [296]. Cocrystals of ORV and proline (ORV-PRO) and nicotinamide (ORV-NCT) also showed enhanced solubility and dissolution in water [297,298].

A microemulsion of ORV, consisting of ethyl butyrate, Tween 80, PEG400, ascorbic acid, and water, showed improved solubility and stability, and exhibited higher anti-browning effects on fresh-cut lotus root slices than 4-hexylresorcinol [299].

When ORV was allowed to react with hexachlorocyclotriphosphazene, nanoparticles of highly cross-linked and monodispersed polyphosphazene with entrapped ORV (ORV-PPP) were formed [300]. The ORV-PPP complexes were stable and exhibited strong fluorescence against UV light; thus, they have potential uses as fluorescent labeling agents or drug carriers.

## 7. Conclusions

Oxyresveratrol has received tremendous attention in recent years. This review provides a comprehensive and up-to-date summary, covering research on oxyresveratrol from 1955 to May 2021. The occurrence and distribution of oxyresveratrol in the plant kingdom are of chemotaxonomic and biosynthetic importance. Oxyresveratrol can be obtained from several sources, including plants, chemical synthesis, and biotransformation. Several analytical methods for the determination of oxyresveratrol in plant materials and products are also available. Compelling evidence from many biological and pharmacological investigations suggests the enormous therapeutic potential of oxyresveratrol in several diseases. Studies on the pharmacokinetic profiles and delivery systems of oxyresveratrol have provided promising results and offered novel approaches for developing oxyresveratrol as a new therapeutic agent.

## Figures and Tables

**Table 1:** Distribution of oxyresveratrol (**1**) in free form.

Plant Name	Family	Reference
**Gymnosperms**		
*Gnetum africanum* Welw.	Gnetaceae	[18]
*Gnetum cuspidatum* Blume	Gnetaceae	[19]
*Gnetum gnemonoides* Brongn.	Gnetaceae	[20]
*Gnetum hainanse* C.Y. Cheng ex L.K. Fu, Y.F. Yu & M.G. Gilbert	Gnetaceae	[21,22]
*Gnetum montanum* Markgr.	Gnetaceae	[23]
*Gnetum pendulum* C.Y. Cheng	Gnetaceae	[24,25]
**Angiosperms**		
**1. Class: Monocotyledons**		
*Chrysopogon aciculatis* (Retz.) Trin.	Poaceae	[26]
*Schoenocaulon officinale* (Schltdl. & Cham.) A. Gray ex Benth.	Liliaceae	[6]
*Smilax china* L.	Smilacaceae	[27,28,29,30,31,32,33,34,35,36,37]
*Smilax microphylla* C.H.Wright	Smilacaceae	[38]
*Veratrum album* L.	Melanthiaceae	[10]
*Veratrum dahuricum* (Turcz.) Loes.	Melanthiaceae	[39]
*Veratrum grandiflorum* (Maxim. Ex Baker) Loes.	Melanthiaceae	[11]
*Veratrum lobelianum* Bernh.	Melanthiaceae	[40]
*Veratrum maackii* Regel	Melanthiaceae	[41]
**2. Class: Dicotyledons**		
**2.1 Subclass: Apetalae (Monochlamydeae)**		
*Artocarpus dadah* Miq.	Moraceae	[42,43]
*Artocarpus fulvicortex* F.M. Jarret	Moraceae	[44]
*Artocarpus gomezianus* Wall. ex Trécul.	Moraceae	[45,46]
*Artocarpus heterophyllus* Lam.	Moraceae	[47,48,49]
*Artocarpus hirsutus* Lam.	Moraceae	[50]
*Artocarpus hypargyreus* Hance ex Benth.	Moraceae	[51]
*Artocarpus lakoocha* Wall. ex Roxb./*Artocarpus lacucha* Buch.-Ham.	Moraceae	[52,53,54,55,56,57]
*Artocarpus nitidus* subsp. *lingnanensis* (Merr.) F.M. Jarrett	Moraceae	[58]
*Artocarpus rigida* Blume	Moraceae	[43]
*Artocarpus styracifolius* Pierre	Moraceae	[59]
*Artocarpus. thailandicus* C.C. Berg	Moraceae	[60]
*Artocarpus xanthocarpus* Merr.	Moraceae	[61]
*Bagassa guianensis* Aubl.	Moraceae	[62]
*Cudrania cochinchinensis* (Lour.) Kudô & Masam.	Moraceae	[63,64,65]
*Cudrania tricuspidate* (Carrière) Bureau ex Lavallée	Moraceae	[66,67,68,69]
*Maclura cochinchinensis* (Lour.) Corner	Moraceae	[70]
*Maclura pomifera* (Raf.) C.K. Schneid.	Moraceae	[71,72,73]
*Morus alba* L.	Moraceae	[74,75,76,77,78,79,80,81,82,83,84,85,86,87,88,89,90,91,92,93,94,95,96,97,98,99,100,101,102,103,104,105,106,107,108]
*Morus atropurpurea* Roxb.	Moraceae	[92,93,108]
*Morus australis* Poir.	Moraceae	[108,109,110]
*Morus bombycis* Koidz	Moraceae	[93,111]
*Morus cathayana* Hemsl.	Moraceae	[108]
*Morus laevigata* Wall. ex Brandis	Moraceae	[108]
*Morus latifolia* Poir.	Moraceae	[85]
*Morus macroura* Miq.	Moraceae	[112,113]
*Morus multicaulis* Perr.	Moraceae	[108]
*Morus nigra* L.	Moraceae	[14,108,114,115,116]
*Morus rubra* L.	Moraceae	[98]
*Morus wittiorum* Hand.-Mazz.	Moraceae	[117]
*Morus yunnanensis* Koidz.	Moraceae	[118]
**2.2 Subclass: Polypetalae**		
*Caesalpinia furfuracea* (Prain) Hattink	Caesalpiniaceae	[119]
*Cassia garrettiana* (Craib) H.S. Irwin & Barneby	Caesalpiniaceae	[120]
*Glycosmis pentaphylla* (Retz.) DC.	Rutaceae	[121]
*Melaleuca Leucadendron* L.	Myrtaceae	[122]
*Prunus dulcis* (Mill.) D.A. Webb	Rosaceae	[123]
*Pterocarpus marsupium* Roxb.	Fabaceae	[124]
*Spirotropis longifolia* (DC.) Baill.	Fabaceae	[125]
*Tetrastigma hemsleyanum* Diels et Gilg.	Vitaceae	[126]

**Table 2:** Inhibition of tyrosinase and related activities by ORV (**1**).

Model	Reference
Mushroom tyrosinase	[14,34,37,49,61,63,67,77,84,97,109,111,124,128,138,139,141,148,149,150,151,152,153,154,155]
Murine tyrosinase from cell lysates	[128,149]
Human tyrosinase from cell lysates	[128]
Cellular tyrosinase/melanogenesis	[49,61,68,77,97,124,128,138,152,153,154,156,157,158,159]
Hypopigmentation in animals	[49,77,97,159]
Depigmentation in humans	[160]
Anti-browning effect	[78]

**Table 3:** Antioxidant and anti-inflammatory activities of ORV.

Model	Reference
DPPH (1,1-diphenyl-2-picrylhydrazyl radical)	[54,61,75,80,90,94,106,114,124,157,162]
O_2_^• −^ (superoxide anion)	[61,75,80,162,163]
HO^•^ (hydroxyl radical)	[80]
ABTS^• +^ (2,2′-azino-bis-(3-ethylbenzthiazoline-6-sulfonic acid radical)	[61,90,116,157,164]
H_2_O_2_ and NO^•^ (nitric oxide radical)	[90,94,165]
Ferric reducing ability of plasma (FRAP)	[90,164]
Peroxy radical oxygen radical absorbance capacity (ORAC)	[90,164]
Rat liver microsomes	[106]
Rat brain homogenates	[76]
Liposomes	[54]
Cyclooxygenase-1 (COX-1), cyclooxygenase-2 (COX-2), and lipoxygenases	[42,166]
Mouse mammary in organ culture	[42]
Mouse microglial cells and primary mixed glial cultures	[94]
Mouse P19 cells	[167]
Mouse BV-2 microglial cells	[168]
Mouse macrophage RAW 264.7.cells	[47,106,169,170,171]
Rat mast cells	[122]
Rat polymorphonuclear leukocytes (PMNs)	[117]
Rat cortical neurons	[27]
Rat PC12 pheochromocytoma cells	[172]
Human Jurkat leukemic T cells	[93]
Human periodontal ligament (hPDL) cells	[173]
Human lens epithelial cells (HLECs)	[174]
Human primary epidermal keratinocytes	[68]
Human embryonic fibroblast (HEF) cells	[175]
Human chondrocyte cells	[169]
Human breast adenocarcinoma cells	[176]
Human intestinal Caco-2 cells	[177,178]
Human intestinal goblet LS 174T cells	[179,180]
Human microglia cells	[181]
Mouse model of ovalbumin (OVA)-induced allergic airway inflammation	[182]
Mouse model of ethanol-induced ulceration	[183]
Mouse model of dextran sodium sulfate-treated colitis	[171]
Rat model of carrageenan-induced hind-paw edema	[106,169]
Rat model of dextran sulfate sodium-induced colitis	[184]
Molecular docking	[185]

**Table 4:** Comparison of selected biological/pharmacological activities of oxyresveratrol and resveratrol.

Biological/Pharmacological Activity	Oxyresveratrol	Resveratrol	Reference
(1) Antioxidant and anti-inflammatory activities			
DPPH	higher		[61,94]
O_2_^− •^	higher		[61]
H_2_O_2_	higher		[94,165]
NO^•^	higher		[94]
FRAP	higher		[164]
ABTS^+ •^	higher		[61,116,164]
ORAC		higher	[164]
Inhibition of LPS-induced production of NO in BV-2 microglial cells		higher	[18,168]
COX-1, COX-2		higher	[166]
5-Lipoxigenase	higher		[166]
Protection against CCl_4_--induced liver fibrosis in rats	higher		[204]
Inhibition of PAF-induced release of β-glucuronidase in rat polymorphonuclear leukocytes	higher		[117]
(2) Inhibition of tyrosinase or melanogensis			
Mushroom tyrosinase (l-dopa)	higher		[124,138,148]
		higher	[152]
Mushroom tyrosinase (l-tyrosine)	higher		[61,138,151,152]
Murine tyrosinase (l-tyrosine)	higher		[149]
Cellular tyrosinase activity and melanogenesis in B16F0 cells	higher		[128,153]
Melanogenesis in *Streptomyces bikiniensis*	higher		[154]
(3) Neuroprotection			
Prevention of 6-OHDA neurotoxicity (Model of Parkinson’s disease)	higher		[79,196,197]
Prevention of KA neurotoxicity	higher		[199]
Prevention of EtOH neurotoxicity		higher	[41]
(4) Glucose and lipid metabolism			
Inhibition of α-glucosidase	higher		[220]
Stimulation of insulin secretion in MIN 6 cells	higher		[226]
Stimulation of glucokinase activity and expression		higher	[223]
Inhibition of AGE (advanced glycation end-products) formation			
Model of BSA-acrolein	higher		[225]
Model of BSA-glucose	higher		[124]
Model of BSA-methylglyoxal		higher	[225]
Stimulation of PGC-1β-mediated gene expression (Peroxisome proliferator activated receptor gamma co-activator-1β)	higher		[230]
Reduction of Firmicutes to Bacteroidetes ratio (F/B) in feces		higher	[248]
(5) Antimicrobial activities			
Antifungal activity	higher		[125]
Inhibition of *Staphylococcus aureus* (Gram positive)	higher		[244]
Inhibition of quorum-sensing of *Chromobacterium violaceum* (Gram negative)		higher	[247]
(6) Others			
Inhibition of phosphodiesterase-2	higher		[264]
Inhibition of phosphodiesterase-4		higher	[257]

## References

[1] Xu L., Liu C., Xiang W., Chen H., Qin X., Huang X. (2014). Advances in the study of oxyresveratrol. Int. J. Pharmacol..

[2] Lim Y.-H., Kim K.-H., Kim J.-K. (2015). Source, biosynthesis, biological activities and pharmacokinetics of oxyresveratrol. Korean J. Food Sci. Tech..

[3] Jeandet P., Delaunois B., Conreux A., Donnez D., Nuzzo V., Cordelier S., Clément C., Courot E. (2010). Biosynthesis, metabolism, molecular engineering, and biological functions of stilbene phytoalexins in plants. BioFactors.

[4] Wang C., Zhi S., Liu C., Xu F., Zhao A., Wang X., Ren Y., Li Z., Yu M. (2017). Characterization of stilbene synthase genes in mulberry (*Morus atropurpurea*) and metabolic engineering for the production of resveratrol in *Escherichia coli*. J. Agric. Food Chem..

[5] Maneechai S., De-Eknamkul W., Umehara K., Noguchi H., Likhitwitayawuid K. (2012). Flavonoid and stilbenoid production in callus cultures of *Artocarpus lakoocha*. Phytochemistry.

[6] Kanchanapoom T., Suga K., Kasai R., Yamasaki K., Kamel M.S., Mohamed M.H. (2002). Stilbene and 2-arylbenzofuran glucosides from the rhizomes of *Schoenocaulon officinale*. Chem. Pharm. Bull..

[7] Awaad A.S. (2006). Phenolic glycosides of *Juncus acutus*. Chem. Nat. Compd..

[8] Dai L.-M., Tang J., Li H.-L., Shen Y.-H., Peng C.-Y., Zhang W.-D. (2009). A new stilbene glycoside from the n-butanol fraction of *Veratrum dahuricum*. Chem. Nat. Compd..

[9] Yang Z.-G., Matsuzaki K., Takamatsu S., Kitanaka S. (2011). Inhibitory effects of constituents from *Morus alba* var. *multicaulis* on differentiation of 3T3-L1 cells and nitric oxide production in RAW264.7 cells. Molecules.

[10] Aydin T., Cakir A., Kazaz C., Bayrak N., Bayir Y., Taşkesenligil Y. (2014). Insecticidal metabolites from the rhizomes of *Veratrum album* against adults of colorado potato beetle, *Leptinotarsa decemlineata*. Chem. Biodivers..

[11] Hanawa F., Tahara S., Mizutani J. (1992). Antifungal stress compounds from *Veratrum grandiflorum* leaves treated with cupric chloride. Phytochemistry.

[12] Choi S.W., Jang Y.J., Lee Y.J., Leem H.H., Kim E.O. (2013). Analysis of functional constituents in mulberry (*Morus alba* L.) twigs by different cultivars, producing areas, and heat processings. Prev. Nutr. Food Sci..

[13] Hakim E.H., Achmad S.A., Aimi N., Indrayanto G., Kitajima M., Makmur L., Surya M.D., Syah Y.M., Takayama H. (2004). Regioselective glucosylation of oxyresveratrol by cell suspension cultures of *Solanum mammosum*. J. Chem. Res..

[14] Zheng Z.-P., Cheng K.-W., Zhu Q., Wang X.-C., Lin Z.-X., Wang M. (2010). Tyrosinase inhibitory constituents from the roots of *Morus nigra*: A structure-activity relationship study. J. Agric. Food Chem..

[15] Qiu F., Komatsu K., Kawasaki K., Saito K., Yao X., Kano Y. (1996). A novel stilbene glucoside, oxyresveratrol 3′-*O*-β-glucopyranoside, from the root bark of *Morus alba*. Planta Med..

[16] Zhang Z., Jin J., Shi L. (2008). Protective function of *cis*-mulberroside A and oxyresveratrol from Ramulus mori against ethanol-induced hepatic damage. Environ. Toxicol. Pharmacol..

[17] Jung J.-W., Park J.-H., Seo K.-H., Baek Y.-S., Oh E.-J., Lee D.-Y., Lim D.-W., Han D., Baek N.-I. (2015). Isolation and identification of phenolic compounds from the root bark of *Morus alba* L.. J. Appl. Biol. Chem..

[18] Nassra M., Krisa S., Papastamoulis Y., Kapche G.D., Bisson J., André C., Konsman J.-P., Schmitter J.-M., Mérillon J.-M., Waffo-Téguo P. (2013). Inhibitory activity of plant stilbenoids against nitric oxide production by lipopolysaccharide-activated microglia. Planta Med..

[19] Shimokawa Y., Hirasawa Y., Kaneda T., Hadi A.H.A., Morita H. (2012). Cuspidans A and B, two new stilbenoids from the bark of *Gnetum cuspidatum*. Chem. Pharm. Bull..

[20] Shimokawa Y., Akao Y., Hirasawa Y., Awang K., Hadi A.H.A., Sato S., Aoyama C., Takeo J., Shiro M., Morita H. (2010). Gneyulins A and B, stilbene trimers, and noidesols A and B, dihydroflavonol-*C*-glucosides, from the bark of *Gnetum gnemonoides*. J. Nat. Prod..

[21] Huang K.-S., Wang Y.-H., Li R.-L., Lin M. (2000). Five new stilbene dimers from the lianas of *Gnetum hainanense*. J. Nat. Prod..

[22] Huang K.-S., Li R.-L., Wang Y.-H., Lin M. (2001). Three new stilbene trimers from the lianas of *Gnetum hainanense*. Planta Med..

[23] Li X.-M., Lin M., Wang Y.-H., Liu X. (2004). Four new stilbenoids from the lianas of *Gnetum montanum* f. *megalocarpum*. Planta Med..

[24] Li X.-M., Lin M., Wang Y.-H. (2003). Stilbenoids from the lianas of *Gnetum pendulum*. J. Asian Nat. Prod. Res..

[25] Li X.M., Wang Y.H., Lin M. (2001). Gnetupendin C, a New Stilbene Dimer from the Lianas of *Gnetum pendulum*. Chin. Chem. Lett..

[26] Shen C.-C., Cheng J.-J., Lay H.-L., Wu S.-Y., Ni C.-L., Teng C.-M., Chen C.-C. (2012). Cytotoxic apigenin derivatives from *Chrysopogon aciculatis*. J. Nat. Prod..

[27] Ban J.Y., Jeon S.Y., Ngyen T.T.H., Bae K.H., Song K.S., Seong Y.H. (2006). Neuroprotective effect of oxyresveratrol from *Smilacis Chinae* rhizome on amyloid β protein (25–35)-induced neurotoxicity in cultured rat cortical neurons. Biol. Pharm. Bull..

[28] Jeon S.-Y., Kwon S.-H., Seong Y.-H., Bae K., Hur J.-M., Lee Y.-Y., Suh D.-Y., Song K.-S. (2007). β-secretase (BACE1)-inhibiting stilbenoids from Smilax Rhizoma. Phytomedicine.

[29] Shao B., Guo H., Cui Y., Liu A., Yu H., Guo H., Xu M., Guo D. (2007). Simultaneous determination of six major stilbenes and flavonoids in *Smilax china* by high performance liquid chromatography. J. Pharm. Biomed. Anal..

[30] Huang H.-L., Guo D.-A., Li P. (2008). Determination of stilbenoids in *Smilax china* L. by HPLC. Chin. J. New Drugs.

[31] Ban J.Y., Cho S.O., Choi S.-H., Ju H.S., Kim J.Y., Bae K., Song K.-S., Seong Y.H. (2008). Neuroprotective effect of *Smilacis chinae* rhizome on NMDA-induced neurotoxicity in vitro and focal cerebral ischemia in vivo. J. Pharmacol. Sci..

[32] Shao B., Guo H.-Z., Guo D.-A. (2009). Flavonoids and stilbenes from *Smilax china*. Chin. Tradit. Herb. Drugs.

[33] Chen L., Yin H., Lan Z., Ma S., Zhang C., Yang Z., Li P.A., Lin B. (2011). Anti-hyperuricemic and nephroprotective effects of *Smilax china* L.. J. Ethnopharmacol..

[34] Liang C., Lim J.-H., Kim S.-H., Kim D.-S. (2012). Dioscin: A synergistic tyrosinase inhibitor from the roots of *Smilax china*. Food Chem..

[35] Kim K.-M., Suh J.-W., Yang S.-H., Kim B.-R., Park T.-S., Shim S.-M. (2014). *Smilax china* root extract detoxifies nicotine by reducing reactive oxygen species and inducing CYP2A6. J. Food Sci..

[36] Yoon S.-R., Yang S.-H., Sub J.-W., Shim S.-M. (2014). Fermentation of *Smilax china* root by *Aspergillus usami* and *Saccharomyces cerevisiae* promoted concentration of resveratrol and oxyresveratrol and the free-radical scavenging activity. J. Sci. Food Agric..

[37] Wang W.-X., Qian J.-Y., Wang X.-J., Jiang A.-P., Jia A.-Q. (2014). Anti-HIV-1 activities of extracts and phenolics from *Smilax china* L.. Pakistan J. Pharm. Sci..

[38] Liu L.-S., Huang H.-L., Liu R.-H., Ren G., Shao F., Ye Y.-H., Lin T. (2013). A new lyoniresinol derivative from *Smilax microphylla*. Nat. Prod. Comm..

[39] Cong Y., Guo J.-G., Liu J. (2013). Two new chemical constituents of *Veratrum dahuricum* (Turcz.) Loes. f. Helv. Chim. Acta.

[40] Tabanca N., Ali Z., Bernier U.R., Epsky N., Nalbantsoy A., Khan I.A., Ali A. (2018). Bioassay-guided isolation and identification of *Aedes aegypti* larvicidal and biting deterrent compounds from *Veratrum lobelianum*. Open Chem..

[41] Wu Y., Li S., Liu J., Liu X., Ruan W., Lu J., Liu Y., Lawson T., Shimoni O., Lovejoy D.B. (2018). Stilbenes from *Veratrum maackii* Regel protect against ethanol-induced DNA damage in mouse cerebellum and cerebral cortex. ACS Chem. Neurosci..

[42] Su B.-N., Cuendet M., Hawthorne M.E., Kardono L.B.S., Riswan S., Fong H.H.S., Mehta R.G., Pezzuto J.M., Kinghorn A.D. (2002). Constituents of the bark and twigs of *Artocarpus dadah* with cyclooxygenase inhibitory activity. J. Nat. Prod..

[43] Suhartati T., Yandria, Suwandp J.F., Hadi S. (2010). In vitro and in vivo antiplasmodial activity of oxyresveratrol and artonin E isolated from two *Artocarpus* plants in Indonesia. Orient. J. Chem..

[44] Shah M.K.K., Sirat H.M., Jamil S. (2016). Cholinesterase inhibitors from heartwood of *Artocarpus fulvicortex* F. M. Jarret. J. Teknol..

[45] Hakim E.H., Ulinnuha U.Z., Syah Y.M., Ghisalberti E.L. (2002). Artoindonesianins N and O, new prenylated stilbene and prenylated arylbenzofuran derivatives from *Artocarpus gomezianus*. Fitoterapia.

[46] Likhitwitayawuid K., Chaiwiriya S., Sritularak B., Lipipun V. (2006). Antiherpetic flavones from the heartwood of *Artocarpus gomezianus*. Chem. Biodivers..

[47] Fang S.-C., Hsu C.-L., Yen G.-C. (2008). Anti-inflammatory effects of phenolic compounds isolated from the fruits of *Artocarpus heterophyllus*. J. Agric. Food Chem..

[48] Zhai X.-X., Zhong G.-Y., Yao P.-C., Lin Q.-H., Yuan W.-J., Ren G. (2016). Phenolic constituents from roots of *Artocarpus heterophyllus*. Zhong Cao Yao.

[49] Li J., Lin Z., Tang X., Liu G., Chen Y., Zhai X., Huang Q., Cao Y. (2020). Oxyresveratrol extracted from *Artocarpus heterophyllus* Lam. inhibits tyrosinase and age pigments in vitro and in vivo. Food Funct..

[50] Nayak M., Nagarajan A., Majeed M. (2017). Pharmacognostic evaluation of leaf and stem wood extracts of *Artocarpus hirsutus* Lam. Pharmacogn. J..

[51] Qiao X., Zhao T., Wang M., Lei C., Hou A. (2011). Flavonoids from *Artocarpus hypargyreus*. Zhongguo Zhongyao Zazhi.

[52] Likhitwitayawuid K., Sritularak B., Benchanak K., Lipipun V., Mathew J., Schinazi R.F. (2005). Phenolics with antiviral activity from *Millettia erythrocalyx* and *Artocarpus lakoocha*. Nat. Prod. Res..

[53] Maneechai S., Likhitwitayawuid K., Sritularak B., Palanuvej C., Ruangrungsi N., Sirisa-ard P. (2009). Quantitative analysis of oxyresveratrol content in *Artocarpus lakoocha* and ‘Puag-Haad’. Med. Princ. Pract..

[54] Povichit N., Phrutivorapongkul A., Suttajit M., Leelapornpisid P. (2010). Antiglycation and antioxidant activities of oxyresveratrol extracted from the heartwood of *Artocarpus lakoocha* Roxb. Maejo Int. J. Sci. Tech..

[55] Baruah D., Das R.N., Hazarika S., Konwar D. (2015). Biogenic synthesis of cellulose supported Pd(0) nanoparticles using hearth wood extract of *Artocarpus lakoocha* Roxb—A green, efficient andversatile catalyst for Suzuki and Heck coupling in water under microwave heating. Catal. Commun..

[56] Duangdee N., Chamboonchu N., Kongkiatpaiboon S., Prateeptongkum S. (2019). Quantitative 1HNMR spectroscopy for the determination of oxyresveratrol in *Artocarpus lacucha* heartwood. Phytochem. Anal..

[57] Songoen W., Phanchai W., Brecker L., Wenisch D., Jakupec M.A., Pluempanupat W., Schinnerl J. (2021). Highly aromatic flavan-3-ol derivatives from palaeotropical *Artocarpus lacucha* Buch.-Ham possess aadical scavenging and antiproliferative properties. Molecules.

[58] Ti H., Wu P., Lin L., Wei X. (2011). Stilbenes and flavonoids from *Artocarpus nitidus* subsp. *lingnanensis*. Fitoterapia.

[59] Yi W., Peng J., Ren G., Jiang W., Liang J., Yuan W. (2015). Chemical constitutes from root of *Artocarpus styracifolius*. Zhong Yao Cai.

[60] Aneklaphakij C., Bunsupa S., Sirichamorn Y., Bongcheewin B., Satitpatipan V. (2020). Taxonomic notes on the ‘Mahat’ (*Artocarpus lacucha* and *A. thailandicus*, Moraceae) species complex in Thailand. Plants.

[61] Jin Y.-J., Lin C.-C., Lu T.-M., Li J.-H., Chen I.-S., Kuo Y.-H., Ko H.-H. (2015). Chemical constituents derived from *Artocarpus xanthocarpus* as inhibitors of melanin biosynthesis. Phytochemistry.

[62] Royer M., Herbette G., Eparvier V., Beauchêne J., Thibaut B., Stien D. (2010). Secondary metabolites of *Bagassa guianensis* Aubl. wood: A study of the chemotaxonomy of the Moraceae family. Phytochemistry.

[63] Zheng Z.-P., Zhu Q., Fan C.-L., Tan H.-Y., Wang M. (2011). Phenolic tyrosinase inhibitors from the stems of *Cudrania cochinchinensis*. Food Funct..

[64] Chen L., Zhou Q., Li B., Liu S.-J., & Dong J.-X. (2015). A new flavonoid from *Cudrania cochinchinensis*. Nat. Prod. Res..

[65] Lin C.-F., Chen Y.-J., Huang Y.-L., Chiou W.-F., Chiu J.-H., Chen C.-C. (2012). A new auronol from *Cudrania cochinchinensis*. J. Asian Nat. Prod. Res..

[66] Shan W.-G., Shi L.-L., Ying Y.-M., Hou X.-R., Zhan Z.J. (2013). A new prenylated stilbene derivative from the roots of *Cudrania tricuspidate*. J. Chem. Res..

[67] Zheng Z.-P., Tan H.-Y., Chen J., Wang M. (2013). Characterization of tyrosinase inhibitors in the twigs of *Cudrania tricuspidata* and their structure–activity relationship study. Fitoterapia.

[68] Hu S., Zheng Z., Zhang X., Chen F., Wang M. (2015). Oxyresveratrol and trans-dihydromorin from the twigs of *Cudrania tricuspidata* as hypopigmenting agents against melanogenesis. J. Funct. Foods.

[69] Li C.-P., Chang X.-J., Fang L., Yao J.-B., Wang R.-W., Zhan Z.-J., Ying Y.-M.b., Shan W.-G. (2016). A new rumenic acid derivative from the roots of *Cudrania tricuspidate*. Chem. Nat. Compd..

[70] Vongsak B., Jaisamut S., Gonsap K., Parmontree P. (2020). Optimization of *Maclura cochinchinensis* extract as a cosmeceutical component for antioxidant and anti-tyrosinase activities. Key Eng. Mater..

[71] Schultz T.P., Harms W.B., Nicholas D.D. (1995). Durability of angiosperm heartwood: The importance of extractives. Holzforschung.

[72] Djapić N., Djarmati Z., Sneźana F., Jankov R. (2003). A stilbene from the heartwood of *Maclura pomifera*. J. Serb. Chem. Soc..

[73] Barnes R.A., Gerber N.N. (1955). The antifungal agent from osage orange wood. J. Am. Chem. Soc..

[74] Choi S.-J. (2018). Changes in the secondary metabolites and bioactivity of mulberry leaves upon UV-C irradiation. Korean J. Food Preserv..

[75] Oh H., Ko E.-K., Jun J.-Y., Oh M.-H., Park S.-U., Kang K.-H., Lee H.-S., Kim Y.-C. (2002). Hepatoprotective and free radical scavenging activities of prenylflavonoids, coumarin, and stilbene from *Morus alba*. Planta Med..

[76] Jin W., Na M., An R., Lee H., Bae K., Kang S.S. (2002). Antioxidant compounds from twig of *Morus alba*. Nat. Prod. Sci..

[77] Kang T.L., Kwang S.L., Ji H.J., Byoung K.J., Moon Y.H., Hyun P.K. (2003). Inhibitory effects of Ramulus mori extracts on melanogenesis. J. Cosmet. Sci..

[78] Li H., Cheng K.-W., ChO C.-H., He Z., Wang M. (2007). Oxyresveratrol as an antibrowning agent for cloudy apple juices and fresh-cut apples. J. Agric. Food Chem..

[79] Chao J., Yu M.S., Ho Y.-S., Wang M., Chang R.C.-C. (2008). Dietary oxyresveratrol prevents parkinsonian mimetic 6-hydroxydopamine neurotoxicity. Free Rad. Biol. Med..

[80] Zhang Z., Jin J., Shi L. (2009). Antioxidant properties of ethanolic extract from Ramulus mori (Sangzhi). Food Sci. Tech. Int..

[81] Zhang Z.-F., Lv G., Shi L.-G., Pan H., Wu Y.-Z., Fan L. Oxyresveratrol from Ramulus mori attenuates alloxan-induced liver damage in mice. Proceedings of the 2009 3rd International Conference on Bioinformatics and Biomedical Engineering.

[82] Zhang Z., Shi L. (2012). Detection of antioxidant active compounds in Mori Ramulus by HPLC-MS-DPPH. Zhongguo Zhongyao Zazhi.

[83] Choi S.W., Lee Y.J., Ha S.B., Jeon Y.H., Lee D.H. (2015). Evaluation of biological activity and analysis of functional constituents from different parts of mulberry (*Morus alba* L.) tree. J. Korean Soc. Food Sci. Nutr..

[84] Zhang L., Tao G., Chen J., Zheng Z.-P. (2016). Characterization of a new flavone and tyrosinase inhibition constituents from the twigs of *Morus alba* L.. Molecules.

[85] Park S.Y., Jin B.R., Lee Y.R., Kim Y.J., Park J.B., Jeon Y.H., Choi S.W., Kwon O. (2016). Postprandial hypoglycemic effects of mulberry twig and root bark in vivo and in vitro. J. Nutr. Health.

[86] Joung D.-K., Mun S.-H., Choi S.-H., Kang O.-H., Kim S.-B., Lee Y.-S., Zhou T., Kong R., Choi J.-G., Shin D.-W. (2016). Antibacterial activity of oxyresveratrol against methicillin-resistant *Staphylococcus aureus* and its mechanism. Exp. Ther. Med..

[87] Lu H.-P., Jia Y.-N., Peng Y.-L., Yu Y., Sun S.-L., Yue M.-T., Pan M.-H., Zeng L.-S., Xu L. (2017). Oxyresveratrol, a stilbene compound from *Morus alba* L. twig extract active against *Trichophyton rubrum*. Phytother. Res..

[88] Jeon Y.-H., Choi S.-W. (2018). Validation of an analytical method of oxyresveratrol for standardization of Mulberry (*Morus alba* L.) branch extract as a functional ingredient. Korean J. Food Sci. Techol..

[89] Kim J.-H., Kim K.-H., Lee M.-Y., Lim Y.-H., Kim J.-K. (2018). Accumulation of oxyresveratrol in Ramulus mori upon postharvest storage. Korean J. Food Sci. Technol..

[90] Hwang S., Kim J.-K., Kim I.-H., Lim Y.-H. (2018). Inhibitory effect of ethanolic extract of Ramulus mori on adipogenic differentiation of 3T3-L1 cells and their antioxidant activity. Food Biochem..

[91] Iswandana R., Olisia S., Adriyani M., Jufri M.A., Munim A. (2020). Application of Tween 80 and Tween 20 for microwave-assisted extraction of oxyresveratrol from mulberry (*Morus alba* L.) twigs. J. Appl. Pharm. Sci..

[92] Zhou J., Li S., Wang W., Guo X., Lu X., Yan X., Huang D., Wei B., Cao L. (2013). Variations in the levels of mulberroside A, oxyresveratrol, and resveratrol in mulberries in different seasons and during growth. Sci. World J..

[93] Chen Y.-C., Tien Y.-J., Chen C.-H., Beltran F.N., Amor E.C., Wang R.-J., Wu D.-J., Mettling C., Lin Y.-L., Yang W.-C. (2013). *Morus alba* and active compound oxyresveratrol exert anti-inflammatory activity via inhibition of leukocyte migration involving MEK/ERK signaling. BMC Compl. Alternative Med..

[94] Lorenz P., Roychowdhury S., Engelmann M., Wolf G., Horn T.F.W. (2003). Oxyresveratrol and resveratrol are potent antioxidants and free radical scavengers: Effect on nitrosative and oxidative stress derived from microglial cells. Nitric Oxide.

[95] Shi Y.-W., Wang C.-P., Wang X., Zhang Y.-L., Liu L., Wang R.-W., Ye J.-F., Hu L.-S., Kong L.-D. (2012). Uricosuric and nephroprotective properties of Ramulus Mori ethanol extract in hyperuricemic mice. J. Ethnopharmacol..

[96] Ahn E., Lee J., Jeon Y.-H., Choi S.-W., Kim E. (2017). Anti-diabetic effects of mulberry (*Morus alba* L.) branches and oxyresveratrol in streptozotocin-induced diabetic mice. Food Sci. Biotechnol..

[97] Chaita E., Lambrinidis G., Cheimonidi C., Agalou A., Beis D., Trougakos I., Mikros E., Skaltsounis A.-L., Aligiannis N., Ferreira I.C.F.R. (2017). Anti-melanogenic properties of Greek plants. A novel depigmenting agent from *Morus alba* wood. Molecules.

[98] Ayinampudi S.R., Wang Y.-H., Avula B., Smillie T.J., Khan I.A. (2011). Quantitative analysis of oxyresveratrol in different plant parts of *Morus* species and related genera by HPTLC and HPLC. J. Planar Chromat..

[99] Zhou J., Li S.-X., Yan X.-P., Ji M., Guo X.-Y., Huang D., Zheng Q.-Y. (2013). Comparative and correlational studies on contents of stilbenes in different portions of mulberry. Zhongguo Zhongyao Zazhi.

[100] Liu G., Du J., Liu F., Xu H., Chen H., Tang J., Hua Y.-L., Liu G.-R., Cao Y. (2015). The separation of oxyresveratrol from mulberry root and its effect on steady state embedding and inhibition of tyrosinase activity. Mod. Food Sci. Technol..

[101] Alishlah T., Mun’Im A., Jufri M. (2018). Optimization of imidazolium-based ionic liquid-microwave assisted extraction for oxyresveratrol. J. Young Pharm..

[102] Fadhila M., Mun’im A., Jufri M. (2018). Ionic liquid-based microwave-assisted extraction (Il-MAE) of oxyresveratrol from *Morus alba* roots. J. Appl. Pharm. Sci..

[103] Nugraha M.W., Iswandana R., Jufri M. (2020). Preparation, characterization, and formulation of solid lipid nanoparticles lotion from mulberry roots (*Morus alba* L.). Int. J. App. Pharm..

[104] Kim J.-H., Doh E.-J., Lee G. (2020). Quantitative comparison of the marker compounds in different medicinal parts of *Morus alba* L. using high-performance liquid chromatography-diode array detector with chemometric analysis. Molecules.

[105] Zhao T.-T., Wei H., Chen L.-M., Wang Z.-M., Zhang Q.-W., Zhao Z.-B., Yan L.-H. (2017). HPLC Fingerprints of different medicinal parts of *Morus alba* L.. Chin. Pharm. J..

[106] Chung K., Kim B., Lee M., Kim Y., Chung H., Park J., Moon J. (2003). In-vitro and in-vivo anti-inflammatory effect of oxyresveratrol from *Morus alba* L.. J. Pharm. Pharmacol..

[107] Ryu S.Y., Han Y.N., Han B.H. (1988). Monoamine oxidase-A inhibitors from medicinal plants. Arch. Pharm. Res..

[108] Song W., Wang H.-J., Buchell P., Zhang P.-F., Wei D.-Z., Lu Y.-H. (2009). Phytochemical profiles of different mulberry (*Morus* sp.) species from China. J. Agric. Food Chem..

[109] Zheng Z.-P., Tan H.-Y., Wang M. (2012). Tyrosinase inhibition constituents from the roots of *Morus australis*. Fitoterapia.

[110] Ferlinahayatia, Syah Y.M., Juliawaty L.D., Achmad S.A., Hakim E.H., Takayama H., Said I.M., Latip J. (2008). Phenolic constituents from the wood of *Morus australis* with cytotoxic activity. Z. Naturforsch. C.

[111] Kang K.B., Du Kim S., Kim T.B., Jeong E.J., Kim Y.C., Sung J.H., Sung S.H. (2011). Tyrosinase inhibitory constituents of *Morus bombycis* cortex. Nat. Prod. Sci..

[112] Syah Y.M., Achmad S.A., Ghisalberti E.L., Hakim E.H., Iman M.Z.N., Makmur L., Mujahiddin D. (2000). Andalasin A, a new stilbene dimer from *Morus macroura*. Fitoterapia.

[113] Syah Y.M., Achmad S.A., Ghisalberti E.L., Hakim E.H., Makmur L., Soekamto N.H. (2004). A stilbene dimer, andalasin B, from the root trunk of *Morus macroura*. J. Chem Res..

[114] Mazimba O., Majinda R.R.T., Motlhanka D. (2011). Antioxidant and antibacterial constituents from *Morus nigra*. Afr. J. Pharm. Pharmacol..

[115] Xu L., Liu J., Liu C., Wu C., Wang C.-H., Huang X.-Z. (2013). Response surface optimization of ultrasonic-assisted oxyresveratrol extraction from the bark of cultured black mulberry (*Morus nigra* L.). Sep. Sci. Technol..

[116] Abbas G.M., Abdel Bar F.M., Baraka H.N., Gohar A.A., Lahloub M.-F. (2014). A new antioxidant stilbene and other constituents from the stem bark of *Morus nigra* L.. Nat. Prod. Res..

[117] Tan Y., Liu C., Chen R. (2010). Phenolic constituents from stem bark of *Morus wittiorum* and their antiinflammation and cytotoxicity. Zhongguo Zhongyao Zazhi.

[118] Cui X.-Q., Chen R.-Y. (2010). Study on components from stem bark of *Morus yunnanensis*. Zhong Cao Yao.

[119] Siridechakorn I., Cheenpracha S., Ritthiwigrom T., Phakhodee W., Deachathai S., Machan T., Ruankeaw N., Laphookhieo S. (2014). Isopimarane diterpenes and flavan derivatives from the twigs of *Caesalpinia furfuracea*. Phytochem. Lett..

[120] Tewtrakul S., Subhadhirasakul S., Rattanasuwan P., Puripattanvong J. (2007). HIV-1 protease inhibitory substances from *Cassia garrettiana*. Songklanakarin J. Sci. Technol..

[121] Wu Y., Hu X., Yang G.-Z., Mei Z.-N., Chen Y. (2012). Two new flavanols from *Glycosmis pentaphylla*. J. Asian Nat. Prod. Res..

[122] Tsuruga T., Chun Y.-T., Ebizuka Y., Sankawa U. (1991). Biologically active constituents of *Melaleuca leucadendron*: Inhibitors of induced histamine release from rat mast cells. Chem. Pharm. Bull..

[123] Xie L., Bolling B.W. (2014). Characterisation of stilbenes in California almonds (*Prunus dulcis*) by UHPLC–MS. Food Chem..

[124] Deguchi T., Tamai A., Asahara K., Miyamoto K., Miyamoto A., Nomura M., Kawata-Tominaga T., Yoshioka Y. (2019). Anti-tyrosinase and anti-oxidative activities by Asana: The heartwood of *Pterocarpus marsupium*. Nat. Prod. Commun..

[125] Basset C., Rodrigues A.M.S., Eparvier V., Silva M.R.R., Lopes N.P., Sabatier D., Fonty E., Espindol L.S., Stien D. (2012). Secondary metabolites from *Spirotropis longifolia* (DC) Baill and their antifungal activity against human pathogenic fungi. Phytochemistry.

[126] Fu Z.-Q., Huang Z.-H., Lin J., He W.-D., Ji M.-M., Xu W., Fan S.-M. (2015). Chemical constituents in root tuber of *Tetrastigma hemsleyanum* and their anti-oxidative activities. Zhong Cao Yao.

[127] Rivière C., Pawlus A.D., Mérillon J.-M. (2012). Natural stilbenoids: Distribution in the plant kingdom and chemotaxonomic interest in Vitaceae. Nat. Prod. Rep..

[128] Park J., Park J.H., Suh H.-J., Lee I.C., Koh J., Boo Y.C. (2014). Effects of resveratrol, oxyresveratrol, and their acetylated derivatives on cellular melanogenesis. Arch. Dermatol. Res..

[129] Deng H., He X., Xu Y., Hu X. (2012). Oxyresveratrol from Mulberry as a dihydrate. Acta Cryst..

[130] Kitisripanya T., Inyai C., Krittanai S., Likhitwitayawuid K., Sritularak B., Ploypradith P., Tanaka H., Morimoto S., Putalun W. (2017). A monoclonal antibody-based immunoassay for the determination of oxyresveratrol from *Artocarpus lacucha* Buch.-Ham. J. Nat. Med..

[131] Reimann E. (1970). Natürliche polyhydroxystilbene. Die synthese vor oxyresveratrol, piceatannol und rhapontigenin. Tet. Lett..

[132] Reimann E. (1971). Natürliche stilbene, II. Synthese von polyhydroxystilbenen. Justus Liebigs Ann. Chem..

[133] Lee S.K., Nam K.A., Hoe Y.H., Min H.-Y., Kim E.-Y., Ko H., Song S., Lee T., Kim S. (2003). Synthesis and evaluation of cytotoxicity of stilbene analogues. Arch. Pharm. Res..

[134] Sun H.-Y., Xiao C.-F., Cai Y.-C., Chen Y., Wei W., Liu X.-K., Lu Z.-L., Zou Y. (2010). Efficient synthesis of natural polyphenolic stilbenes: Resveratrol, piceatannol and oxyresveratrol. Chem. Pharm. Bull..

[135] Sun H., Xiao C., Wei W., Chen Y., Lu Z., Zou Y. (2010). Synthesis of oxyresveratrol. Chin. J. Org. Chem..

[136] Li Z., Kang S., Chen L., Wang Y., Li J. (2016). Study on the synthesis of oxyresveratrol catalyzed by palladium. Chin. J. Org. Chem..

[137] Maafi M., Al-Qarni M.A. (2018). Φ-order spectrophotokinetic characterisation and quantification of trans-cis oxyresveratrol reactivity, photodegradation and actinometry. Spectrochim. Acta A Mol. Biomol. Spectrosc..

[138] Kim D.H., Kim J.H., Baek S.H., Seo J.H., Kho Y.H., Oh T.K., Lee C.H. (2004). Enhancement of tyrosinase inhibition of the extract of *Veratrum patulum* using cellulase. Biotechnol. Bioeng..

[139] Kim J.-K., Kim M., Cho S.-G., Kim M.-K., Kim S.-W., Lim Y.-H. (2010). Biotransformation of mulberroside A from *Morus alba* results in enhancement of tyrosinase inhibition. J. Ind. Microbiol. Biotechnol..

[140] Park K.-T., Kim J.-K., Lim Y.-H. (2014). Deglycosylation of stilbene glucoside compounds improves inhibition of 3-hydroxy-3-methylglutaryl coenzyme A reductase and squalene synthase activities. Food Sci. Biotechnol..

[141] Kim J.S., You H.J., Kang H.Y., Ji G.E. (2012). Enhancement of the tyrosinase inhibitory activity of Mori Cortex Radicis extract by biotransformation using *Leuconostoc paramesenteroides* PR. Biosci. Biotechnol. Biochem..

[142] Aranda C., Ullrich R., Kiebist J., Scheibner K., del Río J.C., Hofrichter M., Martínez A.T., Gutiérrez A. (2018). Selective synthesis of the resveratrol analogue 4,4′-dihydroxy-*trans*-stilbene and stilbenoids modification by fungal peroxygenases. Catal. Sci. Technol..

[143] Komaikul J., Kitisripanya T., Inyai C., Likhitwitayawuid K., Sritularak B., Tanaka H., Putalun W. (2019). Phytostilbenoid production in white mulberry (*Morus alba* L.) cell culture using bioreactors and simple deglycosylation by endogenous enzymatic hydrolysis. Vitr. Cell. Dev. Biol. Plant.

[144] Komaikul J., Kitisripanya T., Likhitwitayawuid K., Sritularak B., Tanaka H.E., Putalun W. (2019). Improvement of stilbenoid production by 2-hydroxypropyl-β-cyclodextrin in white mulberry (*Morus alba* L.) callus cultures. Nat. Prod. Res..

[145] Inyai C., Boonsnongcheep P., Komaikul J., Sritularak B., Tanaka H., Putalun W. (2019). Alginate immobilization of *Morus alba* L. cell suspension cultures improved the accumulation and secretion of stilbenoids. Bioprocess. Biosyst. Eng..

[146] Inyai C., Yusakul G., Komaikul J., Kitisripanya T., Likhitwitayawuid K., Sritularak B., Putalun W. (2021). Improvement of stilbene production by mulberry *Morus alba* root culture via precursor feeding and co-elicitation. Bioprocess Biosyst. Eng..

[147] Likhitwitayawuid K. (2008). Stilbenes with tyrosinase inhibitory activity. Curr. Sci..

[148] Shin N.-H., Ryu S.Y., Choi E.J., Kang S.-H., Chang I.-M., Min K.R., Kim Y. (1998). Oxyresveratrol as the potent inhibitor on dopa oxidase activity of mushroom tyrosinase. Biochem. Biophys. Res. Comm..

[149] Kim Y.M., Yun J., Lee C.-K., Lee H., Min K.R., Kim Y. (2002). Oxyresveratrol and hydroxystilbene compounds. Inhibitory effect on tyrosinase and mechanism of action. J. Biol. Chem..

[150] Likhitwitayawuid K., Sornsute A., Sritularak B., Ploypradith P. (2006). Chemical transformations of oxyresveratrol (trans-2,4,3′,5′-tetrahydroxystilbene) into a potent tyrosinase inhibitor and a strong cytotoxic agent. Bioorg. Med. Chem. Lett..

[151] Ortiz-Ruiz C.V., Ballesta De Los Santos M., Berna J., Fenoll J., Garcia-Ruiz P.A., Tudela J., Garcia-Canovas F. (2015). Kinetic characterization of oxyresveratrol as a tyrosinase substrate. IUBMB Life.

[152] Wang Y., Hao M.-M., Sun Y., Wang L.-F., Wang H., Zhang Y.-J., Li H.-Y., Zhuang P.-W., Yang Z. (2018). Synergistic promotion on tyrosinase inhibition by antioxidants. Molecules.

[153] Zeng H.-J., Li Q.-Y., Ma J., Yang R., Qu L.-B. (2021). A comparative study on the effects of resveratrol and oxyresveratrol against tyrosinase activity and their inhibitory mechanism. Spectrochim. Acta A Mol. Biomol. Spectrosc..

[154] Kim J.-K., Park K.-T., Lee H.-S., Kim M., Lim Y.-H. (2012). Evaluation of the inhibition of mushroom tyrosinase and cellular tyrosinase activities of oxyresveratrol: Comparison with mulberroside A. J. Enzyme Inhib. Med. Chem..

[155] Wang S., Liu X.-M., Zhang J., Zhang Y.-Q. (2014). An efficient preparation of mulberroside a from the branch bark of mulberry and its effect on the inhibition of tyrosinase activity. PLoS ONE.

[156] Rodboon T., Puchadapirom P., Okada S., Suwannalert P. (2016). Oxyresveratrol from *Artocarpus lakoocha* Roxb. inhibit melanogenesis in B16 melanoma cells through the role of cellular oxidants. Walailak J. Sci. Tech..

[157] Rodboon T., Palipoch S., Okada S., Charoenchon N., Nakornpakdee Y., Suwannalert P. (2020). Oxyresveratrol inhibits cellular tyrosinase-related oxidative stress-induced melanogenesis in B16 melanoma cells. J. Appl. Pharm. Sci..

[158] Panichakul T., Rodboon T., Suwannalert P., Tripetch C., Rungruang R., Boohuad N., Youdee P. (2020). Additive effect of a combination of *Artocarpus lakoocha* and *Glycyrrhiza glabra* extracts on tyrosinase inhibition in melanoma B16 cells. Pharmaceuticals.

[159] Park K.-T., Kim J.-K., Hwang D., Yoo Y., Lim Y.-H. (2011). Inhibitory effect of mulberroside A and its derivatives on melanogenesis induced by ultraviolet B irradiation. Food Chem. Toxicol..

[160] Tengamnuay P., Pengrungruangwong K., Pheansri I., Likhitwitayawuid K. (2006). *Artocarpus lakoocha* heartwood extract as a novel cosmetic ingredient: Evaluation of the in vitro anti-tyrosinase and in vivo skin whitening activities. Int. J. Cosmet. Sci..

[161] Park K.-T., Kim J.-K., Lim Y.-H. (2012). Evaluation on skin irritation and sensitization of oxyresveratrol and oxyresveratrol-3-*O*-glucoside produced by biotransformation of *Morus alba* extract. Korean J. Food Sci. Technol..

[162] Chatsumpun N., Chuanasa T., Sritularak B., Lipipun V., Jongbunprasert V., Ruchirawat S., Ploypradith P., Likhitwitayawuid K. (2016). Oxyresveratrol: Structural modification and evaluation of biological activities. Molecules.

[163] Chatsumpun M., Chuanasa T., Sritularak B., Likhitwitayawuid K. (2011). Oxyresveratrol protects against DNA damage induced by photosensitized riboflavin. Nat. Prod. Comm..

[164] Rodríguez-Bonilla P., Gandía-Herrero F., Matencio A., García-Carmona F., López-Nicolás J.M. (2017). Comparative study of the antioxidant capacity of four stilbenes using ORAC, ABTS+, and FRAP techniques. Food Anal. Methods.

[165] Aftab N., Likhitwitayawuid K., Vieira A. (2010). Comparative antioxidant activities and synergism of resveratrol and oxyresveratrol. Nat. Prod. Res..

[166] Kutil Z., Kvasnicova M., Temml V., Schuster D., Marsik P., Cusimamani E.F., Lou J.-D., Vanek T., Landa P. (2015). Effect of dietary stilbenes on 5-lipoxygenase and cyclooxygenases activities in vitro. Int. J. Food Prop..

[167] Tadtong S., Chatsumpun N., Sritularak B., Jongbunprasert V., Ploypradith P., Likhitwitayawuid K. (2016). Effects of oxyresveratrol and its derivatives on cultured P19-derived neurons. Trop. J. Pharm. Res..

[168] Wang L., Zhao H., Wang L., Tao Y., Du G., Guan W., Liu J., Brennan C., Ho C.-T., Li S. (2020). Effects of selected resveratrol analogues on activation and polarization of lipopolysaccharide-stimulated BV-2 microglial cells. J. Agri. Food Chem..

[169] Wongwat T., Srihaphon K., Pitaksutheepong C., Boonyo W., Pitaksuteepong T. (2020). Suppression of inflammatory mediators and matrix metalloproteinase (MMP)-13 by *Morus alba* stem extract and oxyresveratrol in RAW 264.7 cells and C28/I2 human chondrocytes. J. Tradit. Complement. Med..

[170] Lee H.S., Kim D.H., Hong J.E., Lee J.-Y., Kim E.J. (2015). Oxyresveratrol suppresses lipopolysaccharide-induced inflammatory responses in murine macrophages. Hum. Exp. Toxicol..

[171] Hwang D., Jo H., Kim J.-K., Lim Y.-H. (2017). Oxyresveratrol-containing Ramulus mori ethanol extract attenuates acute colitis by suppressing inflammation and increasing mucin secretion. J. Funct. Foods.

[172] Hur J., Kim S., Lee P., Lee Y.M., Choi S.Y. (2013). The protective effects of oxyresveratrol imine derivative against hydrogen peroxide-induced cell death in PC12 cells. Free Rad. Res..

[173] Phoolcharoen W., Sooampon S., Sritularak B., Likhitvitayawuid K., Kuvatanasuchati J., Pavasant P. (2013). Anti-periodontal pathogen and anti-inflammatory activities of oxyresveratrol. Nat. Prod. Comm..

[174] Hu X., Liang Y., Zhao B., Wang Y. (2019). Oxyresveratrol protects human lens epithelial cells against hydrogen peroxide-induced oxidative stress and apoptosis by activation of Akt/HO-1 pathway. J. Pharm. Sci..

[175] Heo J.-I., Kim J.-H., Lee J.-M., Kho Y.-J., Lim S.S., Park J.-B., Kim J., Kim S.C., Lee J.-Y. (2016). FOXO3a Activation by oxyresveratrol of *Morus bombycis* Koidzumi extract mediates antioxidant activity. Anim. Cells Syst..

[176] Wei J., Chen J.-R., Pais E.M., Wang T.-Y., Miao L., Li L., Li L.-Y., Qiu F., Hu L.-M., Gao X.-M. (2017). Oxyresveratrol is a phytoestrogen exerting anti-inflammatory effects through NF-κB and estrogen receptor signaling. Inflammation.

[177] Jo H., Hwang D., Kim J.-K., Lim Y.-H. (2017). Oxyresveratrol improves tight junction integrity through the PKC and MAPK signaling pathways in Caco-2 cells. Food Chem. Toxicol..

[178] Hwang D., Jo H., Hwang S., Kim J.-K., Kim I.-H., Lim Y.-H. (2017). Conditioned medium from LS 174T goblet cells treated with oxyresveratrol strengthens tight junctions in Caco-2 cells. Biomed. Pharmacother..

[179] Hwang D., Jo H., Ma S.-H., Lim Y.-H. (2018). Oxyresveratrol stimulates mucin production in an NAD+-dependent manner in human intestinal goblet cells. Food Chem. Toxicol..

[180] Yeom J., Ma S., Lim Y.-H. (2020). Oxyresveratrol induces autophagy via the ER stress signaling pathway, and oxyresveratrol-induced autophagy stimulates MUC2 synthesis in human goblet. Antioxidants.

[181] Hankittichai P., Lou H.J., Wikan N., Smith D.R., Potikanond S., Nimlamool W. (2020). Oxyresveratrol inhibits IL-1β-induced inflammation via suppressing AKT and ERK1/2 activation in human microglia, HMC3. Int. J. Mol. Sci..

[182] Ashraf M.I., Shahzad M., Shabbir A. (2015). Oxyresveratrol ameliorates allergic airway inflammation via attenuation of IL-4, IL-5, and IL-13 expression levels. Cytokine.

[183] Aziz R.S., Siddiqua A., Shahzad M., Shabbir A., Naseem N. (2019). Oxyresveratrol ameliorates ethanol-induced gastric ulcer via downregulation of IL-6, TNF-α NF-ĸB, and COX-2 levels, and upregulation of TFF-2 levels. Biomed. Pharmacother..

[184] Yeom J., Ma S., Kim J.-K., Lim Y.-H. (2021). Oxyresveratrol ameliorates dextran sulfate sodium-induced colitis in rats by suppressing inflammation. Molecules.

[185] Zhang Y., Zhao Z., Chen H., Fu Y., Wang W., Li Q., Li X., Wang X., Fan G., Zhang Y. (2021). The underlying molecular mechanisms involved in traditional Chinese medicine *Smilax china* L. for the treatment of pelvic inflammatory disease. Evid-Based Compl. Alt. Med..

[186] Radapong S., Sarker S.D., Ritchie K.J. (2020). Oxyresveratrol possesses DNA damaging activity. Molecules.

[187] Andrabi S.A., Spina M.G., Lorenz P., Ebmeyer U., Wolf G., Horn T.F.W. (2004). Oxyresveratrol (trans-2,3′,4,5′-tetrahydroxystilbene) is neuroprotective and inhibits the apoptotic cell death in transient cerebral ischemia. Brain Res..

[188] Breuer C., Wolf G., Andrabi S.A., Lorenz P., Horn T.F.W. (2006). Blood-brain barrier permeability to the neuroprotectant oxyresveratrol. Neurosci. Lett..

[189] Weber J.T., Lamont M., Chibrikova L., Fekkes D., Vlug A., Lorenz P., Kreutzmann P., Slemmer J.E. (2012). Potential neuroprotective effects of oxyresveratrol against traumatic injury. Eur. J. Pharmacol..

[190] Kushwaha P., Singh V., Somvanshi P., Bhardwaj T., Barreto G.E., Ashraf G.M., Mishra B.N., Chundawat R.S., Haque S. (2021). Identification of new BACE1 inhibitors for treating Alzheimer’s disease. J. Mol. Model..

[191] Choi B., Kim S., Jang B.-G., Kim M.-J. (2016). Piceatannol, a natural analogue of resveratrol, effectively reduces beta-amyloid levels via activation of alpha-secretase and matrix metalloproteinase-9. J. Funct. Foods.

[192] Rahman M.A., Cho Y., Nam G., Rhim H. (2021). Antioxidant compound, oxyresveratrol, inhibits app production through the AMPK/ULK1/mTOR-mediated autophagy pathway in mouse cortical astrocytes. Antioxidants.

[193] Sangsen Y., Sooksawate T., Likhitwitayawuid K., Sritularak B., Wiwattanapatapee R. (2018). A Self-microemulsifying formulation of oxyresveratrol prevents amyloid beta protein-induced neurodegeneration in mice. Planta Med..

[194] Rahman M.A., Bishayee K., Sadra A., Huh S.-O. (2017). Oxyresveratrol activates parallel apoptotic and autophagic cell death pathways in neuroblastoma cells. Biochim. Biophys. Acta Gen. Subj..

[195] Hasriadi, Limpeanchob N. (2018). In vitro cytotoxicity of *Artocarpus lakoocha* aqueous extract and oxyresveratrol in SH-SY5Y cells. J. Phys. Conf. Ser..

[196] Shah A., Chao J., Legido-Quigley C., Chang R.C.-C. (2021). Oxyresveratrol exerts ATF4- and Grp78-mediated neuroprotection against endoplasmic reticulum stress in experimental Parkinson’s disease. Nutr. Neurosci..

[197] Shah A., Ho Y.S., Ng K.M., Wang M., Legido-Quigley C., Chang R.C.C. (2020). Neuroprotective effects of oxyresveratrol on 6-hydroxydopamine on medial forebrain bundles in a rat model of Parkinson disease: Abridged secondary publication. Hong Kong Med. J..

[198] Rodsiri R., Benya-aphikul H., Teerapattarakan N., Wanakhachornkrai O., Boonlert W., Tansawat R., Wiwattanapatapee R., Sritularak B., Likhitwitayawuid K. (2020). Neuroprotective effect of oxyresveratrol in rotenone-induced Parkinsonism rats. Nat. Prod. Commun..

[199] Lee H.-J., Feng J.-H., Sim S.-M., Lim S.-S., Lee J.-Y., Suh H.-W. (2019). Effects of resveratrol and oxyresveratrol on hippocampal cell death induced by kainic acid. Anim. Cell. Syst..

[200] Du H., Ma L., Chen G., Li S. (2018). The effects of oxyresveratrol abrogates inflammation and oxidative stress in rat model of spinal cord injury. Mol. Med. Rep..

[201] Choi H.Y., Lee J.-H., Jegal K.H., Cho I.J., Kim Y.W., Kim S.C. (2016). Oxyresveratrol abrogates oxidative stress by activating ERK-Nrf2 pathway in the liver. Chem. Biol. Interact..

[202] Hyrsova L., Vanduchova A., Dusek J.A., Smutny T., Carazo A., Maresova V., Trejtnar F., Barta P., Anzenbacher P., Dvorak Z. (2019). *Trans*-resveratrol, but not other natural stilbenes occurring in food, carries the risk of drug-food interaction via inhibition of cytochrome P450 enzymes or interaction with xenosensor receptors. Toxicol. Lett..

[203] Lee J.-H., Baek S.Y., Jang E.J., Ku S.K., Kim K.M., Ki S.H., Kim C.-E., Park K.I., Kim S.C., Kim Y.W. (2018). Oxyresveratrol ameliorates nonalcoholic fatty liver disease by regulating hepatic lipogenesis and fatty acid oxidation through liver kinase B1 and AMP-activated protein kinase. Chem. Biol. Interact..

[204] Yang G., Zhan J., Yang Y., Yuan L., Wang P., Ho C.-T., Li S. (2021). Inhibitory effects of oxyresveratrol on ERK and Smad1/2 phosphorylation and HSC activation in preventing carbon tetrachloride-induced rat liver fibrosis. Food Sci. Hum. Well..

[205] Lee E.H., Park K.-I., Kim K.-Y., Lee J.-H., Jang E.J., Ku S.K., Kim S.C., Suk H.Y., Park J.Y., Baek S.Y. (2019). Liquiritigenin inhibits hepatic fibrogenesis and TGF-β1/Smad with Hippo/YAP signal. Phytomedicine.

[206] Abid M., Shahani M.Y., Hameed A., Khan S., Sikandar M., Ali H. (2020). Relationship of superoxide dismutase and glutathione peroxidase activities and oxyresveratrol in isoniazid induced hepatotoxicity in experimental model in mice. Pakistan J. Med. Health Sci..

[207] Jia Y.-N., Lu H.-P., Peng Y.-L., Zhang B.-S., Gong X.-B., Su J.C., Zhou Y., Pan M.-H., Xu L. (2018). Oxyresveratrol prevents lipopolysaccharide/D-galactosamine-induced acute liver injury in mice. Int. Immunopharmacol..

[208] Jia Y.-N., Peng Y.-L., Zhao Y.-P., Cheng X.-F., Zhou Y., Chai C.-L., Zeng L.-S., Pan M.-H., Xu L. (2019). Comparison of the hepatoprotective effects of the three main stilbenes from mulberry twigs. J. Agric. Food Chem..

[209] Abdel Bar F.M., Abbas G.M., Gohar A.A., Lahloub M.-F.I. (2020). Antiproliferative activity of stilbene derivatives and other constituents from the stem bark of *Morus nigra* L.. Nat. Prod. Res..

[210] van den Brand A.D., Villevoye J., Nijmeijer S.M., van den Berg M., van Duursen M.B.M. (2019). Anti-tumor properties of methoxylated analogues of resveratrol in malignant MCF-7 but not in non-tumorigenic MCF-10A mammary epithelial cell lines. Toxicology.

[211] Sunilkumar D., Drishya G., Chandrasekharan A., Shaji S.K., Bose C., Jossart J., Perry J.J.P., Mishra N., Kumar G.B., Nair B.G. (2020). Oxyresveratrol drives caspase-independent apoptosis-like cell death in MDA-MB-231 breast cancer cells through the induction of ROS. Biochem. Pharmacol..

[212] Liu D., Kim D.-H., Park J.-M., Na H.-K., Surh Y.-J. (2009). Piceatannol inhibits phorbol ester-induced NF-κB activation and COX-2 expression in cultured human mammary epithelial cells. Nutr. Cancer.

[213] Lv T., Jian Z., Li D., Ao R., Zhabg X., Yu B. (2020). Oxyresveratrol induces apoptosis and inhibits cell viability via inhibition of the STAT3 signaling pathway in Saos-2 cells. Mol. Med. Rep..

[214] Yang Y., Zhang G., Li C., Wang S., Zhu M., Wang J., Yue H., Ma X., Zhen Y., Shu X. (2019). Metabolic profile and structure-activity relationship of resveratrol and its analogs in human bladder cancer cells. Cancer Manag. Res..

[215] Sintuyanon N., Phoolcharoen W., Pavasant P., Sooampon S. (2017). Resveratrol demonstrated higher antiproliferative and antiangiogenic efficacy compared with oxyresveratrol on head and neck squamous cell carcinoma cell lines. Nat. Prod. Commun..

[216] Liu Y., Ren W., Bai Y., Wan L., Sun X., Liu Y., Xiong W., Zhang Y.-Y., Zhou L. (2018). Oxyresveratrol prevents murine H22 hepatocellular carcinoma growth and lymph node metastasis via inhibiting tumor angiogenesis and lymphangiogenesis. J. Nat. Med..

[217] Singh M., Tripathi M., Singh A., Azad C., Gambhir I., Kumar B., Purohit S. (2018). A therapeutic approach to target mitochondrial dysfunction using molecular docking studies: Screening of natural drugs for oral carcinoma. Pharmacogn. Mag..

[218] Liu T., Liu M., Guo Q., Liu Y., Zhao Y., Wu Y., Sun B., Wang Q., Liu J., Han J. (2020). Investigation of binary and ternary systems of human serum albumin with oxyresveratrol/piceatannol and/or mitoxantrone by multipectroscopy, molecular docking and cytotoxicity evaluation. J. Mol. Liq..

[219] Li R., Song Y., Ji Z., Li L., Zhou L. (2020). Pharmacological biotargets and the molecular mechanisms of oxyresveratrol treating colorectal cancer: Network and experimental analyses. BioFactors.

[220] He H., Lu Y.-H. (2013). Comparison of inhibitory activities and mechanisms of five mulberry plant bioactive components against α-glucosidase. J. Agric. Food Chem..

[221] Wongon M., Limpeanchob N. (2020). Inhibitory effect of *Artocarpus lakoocha* Roxb and oxyresveratrol on α-glucosidase and sugar digestion in Caco-2 cells. Heliyon.

[222] Wongon M., Limpeanchob N. (2021). *Artocarpus lacucha* extract and oxyresveratrol inhibit glucose transporters in human intestinal Caco-2 cells. Planta Med..

[223] He H., Yu W.-G., Yang J.-P., Ge S., Lu Y.-H. (2016). Multiple comparisons of glucokinase activation mechanisms of five mulberry bioactive ingredients in hepatocyte. J. Agric. Food Chem..

[224] Yoon J., Lee H., Chang H.B., Choi H., Kim Y.S., Rho Y.K., Seong S., Choi D.H., Park D., Ku B. (2014). DW1029M, a novel botanical drug candidate, inhibits advanced glycation end-product formation, rat lens aldose reductase activity, and TGF-β1 signaling. Am. J. Physiol. Renal Physiol..

[225] Wang W., Yang R., Yao H., Wu Y., Pan W., Jia A.-Q. (2019). Inhibiting the formation of advanced glycation end-products by three stilbenes and the identification of their adducts. Food Chem..

[226] Zheng Y.-C., He H., Wei X., Ge S., Lu Y.-H. (2016). Comparison of regulation mechanisms of five mulberry ingredients on insulin secretion under oxidative stress. J. Agric. Food Chem..

[227] Park S.-Y., Jin B., Shin J.-H., Adisakwattana S., Kwon O. (2017). Standardized Mori ramulus extract improves insulin secretion and insulin sensitivity in C57BLKS/J db/db mice and INS-1 cells. Biomed. Pharmacother..

[228] Tan H.-Y., Tse I.M.Y., Li E.T.S., Wang M. (2015). Inhibitory effects of oxyresveratrol and cyanomaclurin on adipogenesis of 3T3-L1 cells. J. Funct. Foods.

[229] Pavlović N., Đanić M., Stanimirov B., Goločorbin-Kon S., Stankov K., Lalić-Popović M., Mikov M. (2019). In silico discovery of resveratrol analogues as potential agents in treatment of metabolic disorders. Curr. Pharm. Des..

[230] Uchitomi R., Nakai S., Matsuda R., Onishi T., Miura S., Hatazawa Y., Kamei Y. (2019). Genistein, daidzein, and resveratrols stimulate PGC-1β-mediated gene expression. Biochem. Biophys. Rep..

[231] Jo S.-P., Kim J.-K., Lim Y.-H. (2014). Antihyperlipidemic effects of stilbenoids isolated from *Morus alba* in rats fed a high-cholesterol diet. Food Chem. Toxicol..

[232] Hwang D., Jo S.-P., Lee J., Kim J.-K., Kim K.-H., Lim Y.-H. (2015). Antihyperlipidaemic effects of oxyresveratrol containing *Ramulus mori* ethanol extract in rats fed a high-cholesterol diet. J. Funct. Foods.

[233] Choi J.H., Song N.-J., Lee A.R., Lee D.H., Seo M.-J., Kim S., Chang S.-H., Yang D.K., Hwang Y.-J., Hwang K.-A. (2019). Oxyresveratrol increases energy expenditure through Foxo3a-mediated Ucp1 induction in high-fat-diet-induced obese mice. Int. J. Mol. Sci..

[234] Tan H.Y., Tse I.M.Y., Li E.T.S., Wang M. (2017). Oxyresveratrol supplementation to C57bl/6 mice fed with a high-fat diet ameliorates. Nutrients.

[235] Pan M.-H., Koh Y.-C., Lee T.-L., Wang B., Chen W.-K., Nagabhushanam K., Ho C.-T. (2019). Resveratrol and oxyresveratrol activate thermogenesis via different transcriptional coactivators in high-fat diet-induced obese Mice. J. Agric. Food Chem..

[236] Park J.-E., Lee G.-H., Kim J., Choi S.-W., Kim E. (2020). Anti-obesity effect of Ramulus mori extracts and stilbenes in high fat diet-fed C57BL/6J mouse. J. Nutr. Health.

[237] Chuanasa T., Phromjai J., Lipipun V., Likhitwitayawuid K., Suzuki M., Pramyothin P., Hattori M., Shiraki K. (2008). Anti-herpes simplex virus (HSV-1) activity of oxyresveratrol derived from Thai medicinal plant: Mechanism of action and therapeutic efficacy on cutaneous HSV-1 infection in mice. Antiviral Res..

[238] Lipipun V., Sasivimolphan P., Yoshida Y., Daikoku T., Sritularak B., Ritthidej G., Likhitwitayawuid K., Pramyothin P., Hattori M., Shiraki K. (2011). Topical cream-based oxyresveratrol in the treatment of cutaneous HSV-1 infection in mice. Antiviral Res..

[239] Sasivimolphan P., Lipipun V., Ritthidej G., Chitphet K., Yoshida Y., Daikoku T., Sritularak B., Likhitwitayawuid K., Pramyothin P., Hattori M. (2012). Microemulsion-based oxyresveratrol for topical treatment of Herpes Simplex Virus (HSV) infection: Physicochemical properties and efficacy in cutaneous HSV-1 infection in mice. AAPS PharmSciTech.

[240] Sasivimolphan P., Lipipun V., Likhitwitayawuid K., Takemoto M., Pramyothin P., Hattori M., Shiraki K. (2009). Inhibitory activity of oxyresveratrol on wild-type and drug-resistant varicella-zoster virus replication in vitro. Antiviral Res..

[241] Galindo I., Hernáez B., Berná J., Fenoll J., Cenis J.L., Escribano J.M., Alonso C. (2011). Comparative inhibitory activity of the stilbenes resveratrol and oxyresveratrol on African swine fever virus replication. Antivir. Res..

[242] Lin S.-C., Chen M.-C., Li S., Lin C.-C., Wang T.T. (2017). Antiviral activity of nobiletin against chikungunya virus in vitro. Antivir. Ther..

[243] Wu J., Fan Y., Wang X., Jiang X., Zou J., Huang R. (2020). Effects of the natural compound, oxyresveratrol, on the growth of *Streptococcus mutans*, and on biofilm formation, acid production, and virulence gene expression. Eur. J. Oral Sci..

[244] Zakova T., Rondevaldova J., Bernardos A., Landa P., Kokoska L. (2018). The relationship between structure and in vitro antistaphylococcal effect of plant-derived stilbenes. Acta Microbiol. Immunol. Hung..

[245] Joung D.-K., Choi S.-H., Kang O.-H., Kim S.-B., Mun S.-H., Seo Y.-S., Kang D.-H., Gong R., Shin D.-W., Kim Y.-C. (2015). Synergistic effects of oxyresveratrol in conjunction with antibiotics against methicillin-resistant *Staphylococcus aureus*. Mol. Med. Rep..

[246] Lee J.-H., Kim Y.-G., Raorane C.J., Ryu S.Y., Shim J.-J., Lee J. (2019). The anti-biofilm and anti-virulence activities of *trans*-resveratrol and oxyresveratrol against uropathogenic *Escherichia coli*. Biofouling.

[247] Sheng J.-Y., Chen T.-T., Tan X.-J., Chen T., Jia A.-Q. (2015). The quorum-sensing inhibiting effects of stilbenoids and their potential structure-activity relationship. Bioorg. Med. Chem. Lett..

[248] Jaimes J.D., Jarosova V., Vesely O., Mekadim C., Mrazek J., Marsik P., Killer J., Smejkal K., Kloucek P., Havlik J. (2019). Effect of selected stilbenoids on human fecal microbiota. Molecules.

[249] Jarosova V., Vesely O., Marsik P., Jaimes J.D., Smejkal K., Kloucek P., Havlik J. (2019). Metabolism of stilbenoids by human faecal microbiota. Molecules.

[250] Ratanabanangkoon K., Poopyruchpoog N., Charccnsiri K. (1976). (1976) A preliminary study on the antifungal activity of 2,4,3′,5′-tetrahydroxystilbene on dermatophytes. J. Sci. Soc. Thail..

[251] Perrot T., Schwartz M., Saiag F., Salzet G., Dumarçay S., Favier F., Gérardin P., Girardet J.-M., Sormani R., Morel-Rouhier M. (2018). Fungal glutathione transferases as tools to explore the chemical diversity of amazonian wood extractives. ACS Sustain. Chem. Eng..

[252] Kim S., Lee D.G. (2018). Oxyresveratrol-induced DNA cleavage triggers apoptotic response in *Candida albicans*. Microbiology.

[253] Passos C.L.A., Ferreira C., Soares D.C., Saraiva E.M. (2015). Leishmanicidal effect of synthetic *trans*-resveratrol analogs. PLoS ONE.

[254] Janthasoot W., Ratanabanangkoon K., Chudapongse P. (1977). Inhibition of mitochondrial ATPase by 2,4,3′,5′-tetrahydroxystilbene. Chem. Biol. Interact..

[255] Kim S., Ko H., Park J.E., Jung S., Lee S.K., Chun Y.-J. (2002). Design, synthesis, and discovery of novel *trans*-stilbene analogues as potent and selective human cytochrome P450 1B1 inhibitors. J. Med. Chem..

[256] Thu H.N., My L.H.T., Van P.N., Thi H.D. (2020). Bioactivity-guided isolation and identification of xanthine oxidase inhibitors from *Morus alba* bark. J. Adv. Pharm. Res..

[257] Zhao P., Chen S.-K., Cai Y.-H., Lua X., Li Z., Cheng Y.-K., Zhang C., Hu X., He X., Luo H.-B. (2013). The molecular basis for the inhibition of phosphodiesterase-4D by three natural resveratrol analogs. Isolation, molecular docking, molecular dynamics simulations, binding free energy, and bioassay. Biochim. Biophys. Acta.

[258] Liu M., Liu T., Shi Y., Zhao Y., Yan H., Sun B., Wang Q., Wang Z., Han J. (2019). Comparative study on the interaction of oxyresveratrol and piceatannol with trypsin and lysozyme: Binding ability, activity and stability. Food Funct..

[259] Su Y., Sun C., Chen Y., Liu S., Jing N., Li S. (2019). Toxic *trans*-crotonaldehyde in mitochondria intercepted by oxyresveratrol contributing to anticancer. IUBMB Life.

[260] Qiu F., Kano Y., Yao X. (1999). Elucidation of the bioactive constituents in traditional Chinese medicine “Mori Cortex”. Stud. Plan. Sci..

[261] Yin Y., Chen Y., Xu L., Zhao X., Zhuang Q., Tang H., Yang S. (2020). Attenuation of myocardial fibrosis by oxyresveratrol involves mirna-145-mediated cd137/smad/nfatc1 signaling in an atherosclerotic model. Lat. Am. J. Pharm..

[262] Lee J., Kwon G., Park J., Kim J.-K., Lim Y.-H. (2016). SIR-2.1-dependent lifespan extension of *Caenorhabditis elegans* by oxyresveratrol and resveratrol. Exp. Biol. Med..

[263] Matencio A., Guerrero-Rubio M.A., Caldera F., Cecone C., Trotta F., García-Carmona F., López-Nicolás J.M. (2020). Lifespan extension in *Caenorhabditis elegans* by oxyresveratrol supplementation in hyper-branched cyclodextrin-based nanosponges. Int. J. Pharm..

[264] Matencio A., García-Carmona F., López-Nicolás J.M. (2020). Characterization of resveratrol, oxyresveratrol, piceatannol and roflumilast as modulators of phosphodiesterase activity. Study of yeast lifespan. Pharmaceuticals.

[265] Saowakon N., Tansatit T., Wanichanon C., Chanakul W., Reutrakul V., Sobhon P. (2009). Fasciola gigantica: Anthelmintic effect of the aqueous extract of *Artocarpus lakoocha*. Exp. Parasitol..

[266] Rauf A., Imran M., Sulera H.A.R., Ahmad B., Peters D.G., Mubarak M.S. (2017). A comprehensive review of the health perspectives of resveratrol. Food Funct..

[267] Apak R., Ozyurek M., Guclu K., Capanotlu E. (2016). Antioxidant activity/capacity measurement. 1. Classification, physicochemical principles, mechanisms, and electron transfer (ET)-based assays. J. Agric. Food Chem..

[268] Apak R., Ozyurek M., Guclu K., Capanotlu E. (2016). Antioxidant Activity/Capacity Measurement. 2. Hydrogen Atom Transfer (HAT)-Based, Mixed-Mode (Electron Transfer (ET)/HAT), and Lipid Peroxidation Assays. J. Agric. Food Chem..

[269] Lu Y., Wang A., Shi P., Zhang H. (2017). A theoretical study on the antioxidant activity of piceatannol and isorhapontigenin scavenging nitric oxide and nitrogen dioxide radicals. PLoS ONE.

[270] Qiu F., Komatsu K.-I., Saito K.-I., Kawasaki K., Yao X., Kano Y. (1996). Pharmacological properties of traditional medicines (XXI): Analysis of plasma, urine, and bile of rats after oral administration of water extract of Mori Cortex. Nat. Med..

[271] Qiu F., Komatsu K.-I., Saito K.-I., Kawasaki K., Yao X., Kano Y. (1996). Pharmacological properties of traditional medicines. XXII. Pharmacokinetic study of mulberroside A and its metabolites in rat. Biol. Pharm. Bull..

[272] Zhaxi M., Chen L., Li X., Komatsu K., Yao X., Qiu F. (2010). Three major metabolites of mulberroside A in rat intestinal contents and feces. Planta. Med..

[273] Huang H., Zhang J., Chen G., Lu Z., Wang X., Sha N., Shao B., Li P., Guo D. (2008). High performance liquid chromatographic method for the determination and pharmacokinetic studies of oxyresveratrol and resveratrol in rat plasma after oral administration of *Smilax china* extract. Biomed. Chromatogr..

[274] Huang H., Chen G., Lu Z., Zhang J., Guo D. (2009). Distribution study of two constituents in rat after oral administration of *Smilax china* extract. Zhongguo Zhongyao Zazhi.

[275] Huang H., Zhang J., Chen G., Lu Z., Sha N., Guo D. (2009). Simultaneous determination of oxyresveratrol and resveratrol in rat bile and urine by HPLC after oral administration of *Smilax china* extract. Nat. Prod. Comm..

[276] Huang H., Chen G., Lu Z., Zhang J., Guo D. (2010). Identification of seven metabolites of oxyresveratrol in rat urine and bile using liquid chromatography/tandem mass spectrometry. Biomed. Chromatogr..

[277] Bertram R.M., Takemoto J.K., Remsberg C.M., Vega-Villa K.R., Sablani S., Davies N. (2010). High-performance liquid chromatographic analysis: Applications to nutraceutical content and urinary disposition of oxyresveratrol in rats. Biomed. Chromatogr..

[278] Tian F., Wei H., Jia T., Tian H. (2014). An improved highly sensitive method to determine low oxyresveratrol concentrations in rat plasma and its pharmacokinetic application. Biomed. Chromatogr..

[279] Huang H.-L., Huang G.-Y., Liang F., Liu K.-L., Ren G., Shao F., Liu R.-H. (2014). LC-MS/MS method for the determination of oxyresveratrol in rat plasma and its application to pharmacokinetic study. Chin. J. New Drugs.

[280] Chen W., Yeo S.C.M., Elhennawy M.G.A.A., Lin H.-S. (2016). Oxyresveratrol: A bioavailable dietary polyphenol. J. Funct. Foods.

[281] Mei M., Ruan J.-Q., Wu W.-J., Zhou R.-N., Lei J.P.-C., Zhao H.-Y., Yan R., Wang Y.-T. (2012). In vitro pharmacokinetic characterization of mulberroside A, the main polyhydroxylated stilbene in Mulberry (*Morus alba* L.), and its bacterial metabolite oxyresveratrol in traditional oral use. J. Agric. Food Chem..

[282] Hu N., Mei M., Ruan J., Wu W., Wang Y., Yan R. (2014). Regioselective glucuronidation of oxyresveratrol, a natural hydroxystilbene, by human liver and intestinal microsomes and recombinant UGTs. Drug Metab. Pharmacokinet..

[283] Junsaeng D., Anukunwithaya T., Songvut P., Sritularak B., Likhitwitayawuid K., Khemawoot P. (2019). Comparative pharmacokinetics of oxyresveratrol alone and in combination with piperine as a bioenhancer in rats. BMC Compl. Altern. Med..

[284] Rodríguez-Bonilla P., López-Nicolás J.M., García-Carmona F. (2010). Use of reversed phase high pressure liquid cromatography for the physicochemical and thermodynamic characterization of oxyresveratrol/β-cyclodextrin complexes. J. Chromatogr. B.

[285] Yu Y., Feng W., Zhang Y., Wang C., Zhao S. (2013). Preparation of hydroxypropyl cyclodextrin inclusion of oxyresveratrol/pterostilbene. Adv. Mat. Res..

[286] He J., Zheng Z.-P., Zhu Q., Guo F., Chen J. (2017). Encapsulation mechanism of oxyresveratrol by β-cyclodextrin and hydroxypropyl-β-cyclodextrin and computational analysis. Molecules.

[287] Matencio A., García-Carmona F., López-Nicolás J.M. (2017). The inclusion complex of oxyresveratrol in modified cyclodextrins: A thermodynamic, structural, physicochemical, fluorescent and computational study. Food Chem..

[288] He J., Guo F., Lin L., Chen H., Chen J., Cheng Y., Zheng Z.-P. (2019). Investigating the oxyresveratrol β-cyclodextrin and 2-hydroxypropyl-β-cyclodextrin complexes: The effects on oxyresveratrol solution, stability, and antibrowning ability on fresh grape juice. LWT Food Sci. Technol..

[289] Matencio A., Navarro-Orcajada S., Conesa I., Muñoz-Sánchez I., Laveda-Cano L., Cano-Yelo D., García-Carmona F., López-Nicolás J.M. (2020). Evaluation of juice and milk “food models” fortified with oxyresveratrol and β-Cyclodextrin. Food Hydrocoll..

[290] Matencio A., Dhakar N.K., Bessone F., Musso G., Cavalli R., Dianzani C., García-Carmona F., López-Nicolás J.M., Trotta F. (2020). Study of oxyresveratrol complexes with insoluble cyclodextrin based nanosponges: Developing a novel way to obtain their complexation constants and application in an anticancer study. Carbohydr. Polym..

[291] Dhakar N.K., Matencio A., Caldera F., Argenziano M., Cavalli R., Dianzani C., Zanetti M., López-Nicolás J.M., Trotta F. (2019). Comparative evaluation of solubility, cytotoxicity and photostability studies of resveratrol and oxyresveratrol loaded nanosponges. Pharmaceutics.

[292] Sangsen Y., Wiwattanawongsa K., Likhitwitayawuid K., Sritularak B., Wiwattanapatapee R. (2015). Modification of oral absorption of oxyresveratrol using lipid based nanoparticles. Colloids Surf. B.

[293] Sangsen Y., Wiwattanawongsa K., Likhitwitayawuid K., Sritularak B., Graidist P., Wiwattanapatapee R. (2016). Influence of surfactants in self-microemulsifying formulations on enhancing oral bioavailability of oxyresveratrol: Studies in Caco-2 cells and in vivo. Int. J. Pharm..

[294] Sangsen Y., Wiwattanawongsa K., Likhitwitayawuid K., Sritularak B., Wiwattanapatapee R. (2017). Comparisons between a self-microemulsifying system and lipid nanoparticles of oxyresveratrol on the physicochemical properties and Caco-2 cell permeability. Eur. J. Lipid Sci. Technol..

[295] Li J., Wang Q., Liu G., Miao J.-Y., Xie L., Cao Y. (2021). Nanoemulsion improves oral bioavailability and transdermal absorption efficiency of oxyresveratrol. Xiandai Shipin Keji.

[296] Suzuki Y., Muangnoi C., Thaweesest W., Teerawonganan P., Bhuket P.R.N., Titapiwatanakun V., Yoshimura-Fujii M., Sritularak B., Likhitwitayawuid K., Rojsitthisak P. (2019). Exploring novel cocrystalline forms of oxyresveratrol to enhance aqueous solubility and permeability across a cell monolayer. Biol. Pharm. Bull..

[297] Ouiyangkul P., Saithong S., Tantishaiyakul V. (2020). Syntheses and crystal structures of hydrated and anhydrous 1:2 cocrystals of oxyresveratrol and zwitterionic proline. Acta Cryst. E.

[298] Ouiyangkul P., Tantishaiyakul V., Hirun N. (2020). Exploring potential coformers for oxyresveratrol using principal component analysis. Int. J. Pharm..

[299] He J., Zhu Q., Dong X., Pan H., Chen J., Zheng Z. (2017). Oxyresveratrol and ascorbic acid O/W microemulsion: Preparation, characterization, anti-isomerization and potential application as antibrowning agent on fresh-cut lotus root slices. Food Chem..

[300] Hong S., Li J., Huang X., Liu H. (2018). A facile approach to generate cross-linked poly(cyclotriphosphazeneco-oxyresveratrol) nanoparticle with intrinsically fluorescence. J. Inorg. Organomet. Polym. Mat..

